# Nuclear Actin Polymerization Regulates Cell Epithelial‐Mesenchymal Transition

**DOI:** 10.1002/advs.202300425

**Published:** 2023-08-11

**Authors:** William W. Du, Javeria Qadir, Kevin Y. Du, Yu Chen, Nan Wu, Burton B. Yang

**Affiliations:** ^1^ Sunnybrook Research Institute and Department of Laboratory Medicine and Pathobiology University of Toronto Toronto ON M4N3M5 Canada

**Keywords:** actin polymerization, epithelial‐mesenchymal transition, nuclear actin, transcription factor, wound healing

## Abstract

Current studies on actin function primarily rely on cytoplasmic actin due to the absence of cellular models specifically expressing nuclear actin. Here, cell models capable of expressing varying levels of nuclear F/G‐actin are generated and a significant role of nuclear actin in the regulation of epithelial‐mesenchymal transition (EMT) is uncovered. Through immunoprecipitation and mass spectrometry analyses, distinct binding partners for nuclear F‐actin (β‐catenin, SMAD2, and SMAD3) and nuclear G‐actin (MYBBP1A, NKRF, and MYPOP) are investigated, which respectively modulate EMT‐promoting and EMT‐repressing transcriptional events. While nuclear F‐actin promotes EMT with enhanced cell migration, survival, and elongated mesenchymal morphology, nuclear G‐actin represses EMT and related cell activities. Mechanistically, nuclear F‐actin enhances β‐catenin, SMAD2, and SMAD3 expression and stability in the nuclei, while nuclear G‐actin increases MYBBP1A, NKRF, and MYPOP expression and stability in the nuclei. The association between nuclear F/G‐actin and N‐cadherin/E‐cadherin in the cell lines (in vitro), and increased nuclear actin polymerization in the wound healing cells (in vivo) affirm a significant role of nuclear actin in EMT regulation. With evidence of nuclear actin polymerization and EMT during development, and irregularities in disease states such as cancer and fibrosis, targeting nuclear actin dynamics to trigger dysregulated EMT warrants ongoing study.

## Graphical abstract

A cell model is developed that specifically expressed different levels of nuclear F/G‐actin. This has allowed us to specifically increase or decrease the levels of nuclear F‐actin and G‐actin and study their functions. The results revealed a novel role of nuclear actin polymerization and depolymerization in regulating cell EMT by modulating different signaling pathways.

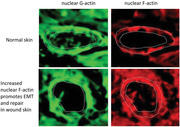

## 1 Introduction

As a major component of cytoskeleton, actin contributes to diverse physiologically relevant processes including cell contractility,^[^ ^1^ ^]^ migration,^[^ ^2^, ^3^ ^]^ mitosis,^[^ ^4^, ^5^ ^]^ endocytosis, and secretion,^[^ ^6^, ^7^ ^]^ cytokinesis,^[^ ^8^ ^]^ and intracellular and extracellular transport.^[^ ^9^, ^10^ ^]^ Actin polymerization, the assembly of monomeric globular actin (G‐actin) into long filamentous actin (F‐actin), and actin depolymerization, the splitting of F‐actin into G‐actin, are the critical events underlying these biological functions.^[^ ^11^, ^12^ ^]^ Due to lacking research tools and cellular models that specifically study the roles of nuclear F/G‐actin excluding the effects of cytoplasmic actin, the reported actin functions have mainly been based on cytoplasmic actin studies, and the current mechanistic knowledge of nuclear actin is limited.^[^ ^13^, ^14^ ^]^

With continuously improving high‐resolution imaging and DNA‐related techniques, the involvement of nuclear actin in DNA‐related processes and its relation to chromatin has been reported in greater detail recently. Nuclear F‐actin and its binding protein, actin‐binding protein 2/3, can bind to the homology‐directed DNA double‐strand breaks and promote its repair.^[^ ^15^, ^16^ ^]^ Increasing evidence indicates that nuclear actin is involved in DNA‐related processes including chromatin remodeling,^[^ ^17^, ^18^ ^]^ transcription,^[^ ^19^ ^]^ and DNA repair.^[^ ^15^, ^16^ ^]^ Nuclear actin dynamics are subject to regulation in diverse physiological processes. It is generally believed that decreased nuclear F‐actin expression represses overall transcription levels,^[^ ^20^ ^]^ whereas increased nuclear F‐actin levels promote particular gene expression.^[^ ^21^ ^]^

Wnt/β‐catenin signaling is a highly conserved pathway that regulates key cellular functions including proliferation, apoptosis, migration, differentiation, and epithelial‐mesenchymal transition (EMT).^[^ ^22^ ^]^ β‐catenin is a core component of the cadherin protein complex, linking cadherins, the adhesion molecules to the cytoskeletal actin filaments (F‐actin).^[^ ^23^ ^]^ β‐catenin links cell‐cell adhesion cadherin complexes to the actin cytoskeleton via interaction with α‐catenin.^[^ ^24^, ^25^, ^26^ ^]^ The nuclear stabilization of β‐catenin is essential for activation of Wnt/β‐catenin signaling.^[^ ^27^ ^]^ Interestingly, F‐actin was found to bind β‐catenin in the nucleus and increase its nuclear stability, which played roles in the regulation of Wnt/β‐catenin signaling.^[^ ^28^ ^]^ As a transcription factor involving multiple signaling pathways, β‐catenin has been functionally implicated in a variety of processes including cell EMT.^[^ ^22^, ^29^ ^]^ However, whether nuclear actin dynamics plays a role in the regulation of EMT is not yet clear.

Actin‐binding compounds, Jasplakinolide (Jasp), Latrunculin B (LatB), and Cytochalasin D (CytD) are widely used to modulate actin dynamics. Actin filament stabilizer Jasp promotes cell actin polymerization, while LatB and CytD induce actin depolymerization. To identify nuclear actin binding partners that function in transcriptional regulation, we found that nuclear F‐actin bound EMT‐promoting transcription factors β‐catenin, SMAD2 and SMAD3, and nuclear G‐actin bound tumor‐suppressing transcription factors MYB Binding Protein 1a (MYBBP1A), NF‐kappa‐B‐repressing factor (NKRF), and MYB‐related transcription factor (MYPOP), resulting in EMT repression. Aberrant nuclear actin polymerization and EMT dysfunction have both been observed in normal cells linking to diseases, such as cancer and fibrosis.^[^ ^30^, ^31^ ^]^ Considering the importance of EMT in embryonic and tissue development, identification of the roles and mechanisms of nuclear actin polymerization in regulating EMT may explore an exciting avenue for further study.

## 2 Results

### 2.1 Nuclear F/G‐Actin Binds Functional Transcription Factors

**Figure 1:**
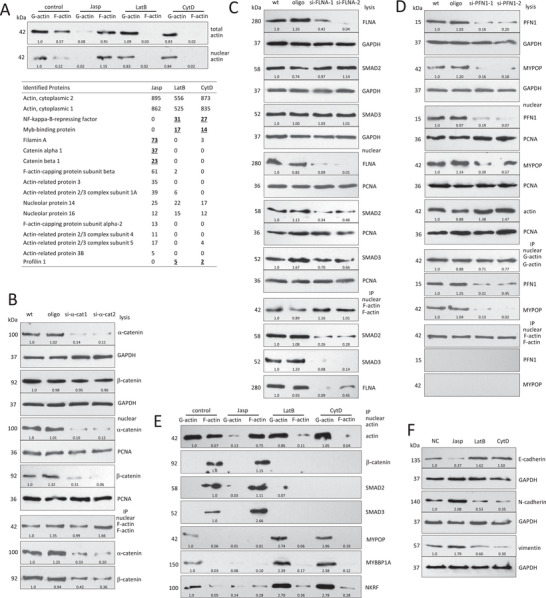
F/G‐actin binds nuclear proteins. A) Upper, HEK 293T cells were cultured in 0.5 µm Jasp, 1 µm LatB, or 10 µm CytD for 1 h, lysed with lysis and F‐actin stabilization buffer 2 (LAS2), and subjected to F/G‐actin fractionation. Western blot showed that Jasp induced actin polymerization, while LatB and CytD induced actin depolymerization. Middle, The above cells were also resuspended in the fractionation buffer, and subjected to nuclear fractionation and F/G‐actin fractionation. Western blot showed that Jasp induced nuclear actin polymerization, while LatB and CytD treatment induced nuclear actin depolymerization. Lower, After cultured in 0.25 µm Jasp, 0.5 µm LatB, or 5 µm CytD for 4 h, HEK 293T cells were subjected to nuclear fractionation. Nuclear extracts were lysed with LAS2, and subjected to immunoprecipitation with antibody against actin. The pulldown products were subjected to mass spectrometry analysis, showing that precipitation of nuclear actin pulled down α‐catenin, β‐catenin, and filamin A (FLNA) in Jasp treated cells, and MYBBP1A, NKRF, and profilin 1 (PFN1) in LatB and CytD treated cells. B) The cells were transfected with α‐catenin siRNAs. Western blot showed that silencing α‐catenin decreased α‐catenin expression, but did not affect β‐catenin expression. Silencing α‐catenin decreased both α‐catenin and β‐catenin in the nuclei. The nuclear extracts were incubated with 25 µL biotin‐X Phalloidin, and subjected to biotin‐labeled Phalloidin pulldown assay. Precipitation of F‐actin pulled down α‐catenin and β‐catenin, and pulled down less α‐catenin (23.4%) and β‐catenin (39.8%) by silencing α‐catenin. C) Western blot showed that silencing FLNA decreased its expression, but did not change SMAD2/SMAD3 levels. Silencing FLNA decreased FLNA, SMAD2, and SMAD3 in the nuclei. Precipitation of nuclear F‐actin pulled down FLNA, SMAD2, and SMAD3, but less FLNA (25.7%), SMAD2 (27.8%) and SMAD3 (10.5%) by silencing FLNA. D) Silencing PFN1 decreased PFN1 and MYPOP levels in cell lysate and nuclei. Precipitation of G‐actin pulled down PFN1 and MYPOP, but less of them by silencing PFN1. E) The nuclear fractions of the above cells were lysed with LAS2 and subjected to F/G‐actin fractionation and immunoprecipitation with antibody against actin. Precipitation of nuclear F‐actin pulled down β‐catenin, SMAD2, SMAD3, and precipitation of nuclear G‐actin pulled down MYBBP1A, NKRF, and MYPOP. F, HEK 293T cells were cultured in 0.1 µm Jasp, 0.2 µm LatB, and 1 µm CytD for 16 h, lysed with lysis buffer, and subjected to Western blot. Jasp treatment repressed E‐cadherin, enhanced N‐cadherin and vimentin proteins. LatB and CytD treatment increased E‐cadherin, decreased N‐cadherin and vimentin levels.

To identify nuclear G/F‐actin binding partners, we precipitated nuclear actin after treating the cells with Jasp, LatB, and CytD. While Jasp enhances actin polymerization, LatB and CytD increase actin depolymerization. After confirming their effects on actin association/dissociation in the nuclei by Western blotting (**Figure** **1**A, upper) and ELISA (Figure S1A, Supporting Information), mass spectrometry was performed that detected α‐catenin, β‐catenin, and filamin A (FLNA) in Jasp treated cells, and MYBBP1A, NKRF, and profilin 1 (PFN1) in LatB or CytD treated samples after nuclear actin precipitation (Figure 1A lower, full list of proteins provided in Table S1, Supporting Information). β‐catenin is known to bind F‐actin via interaction with α‐catenin.^[^ ^25^, ^26^ ^]^ We further analyzed the interaction. After confirming the successful isolation of nuclei (Figure S1B, Supporting Information), nuclear G‐actin and F‐actin were prepared and subjected to anti‐actin antibody precipitation. Anti‐actin antibody precipitated actin, β‐catenin, SMAD2, SMAD3, MYBBP1A, NKRF, and MYPOP. In nuclear G‐actin preparation, MYBBP1A, NKRF, and MYPOP were pulled down, whereas in F‐actin preparation, β‐catenin, SMAD2, and SMAD3 were precipitated (Figure S1B, Supporting Information). To validate whether they interacted with each other in the nuclei, we treated the cells with α‐catenin siRNAs that decreased β‐catenin in the nuclei. Nuclear F‐actin was subjected to biotin‐labeled phalloidin pulldown assay that precipitated nuclear F‐actin, α‐catenin, and β‐catenin. Silencing α‐catenin decreased co‐precipitation of β‐catenin by F‐actin (Figure 1B).

The F‐actin binding protein FLNA has been reported to interact with SMAD.^[^ ^32^ ^]^ Silencing FLNA decreased SMAD2/SMAD3 in the nuclei but not in the total cell lysate (Figure 1C). Precipitation of nuclear F‐actin co‐precipitated FLNA, SMAD2, and SMAD3, but the levels of SMAD2/SMAD3 decreased in the cells treated with FLNA siRNA (Figure 1C). The G‐actin binding protein PFN1 was reported to bind MYPOP.^[^ ^33^, ^34^ ^]^ To test whether this occurred in the nuclei, we transfected cells with PFN1 siRNAs, and found that silencing PFN1 decreased MYPOP in both, the nuclei and the cell lysate (Figure 1D). Precipitation of G‐actin pulled down PFN1 and MYPOP in the nuclei, which was reduced with PFN1 silencing (Figure 1D). Equal amounts of input proteins for Figure 1B‐D (upper panel) are shown by GAPDH and PCNA (also serving as a loading control). MYBBP1A, NKRF, and MYPOP are transcription factors, mainly expressed in the nuclei, with tumor‐suppressing functions.^[^ ^35^, ^36^, ^37^, ^38^ ^]^ We precipitated the nuclear F/G‐actin fractions in the Jasp‐, LatB‐, and CytD‐treated cells, and found that precipitation of nuclear F‐actin pulled down β‐catenin, SMAD2, and SMAD3, while precipitation of nuclear G‐actin pulled down MYBBP1A, NKRF and MYPOP (Figure 1E). The F/G‐actin input in total lysate and nuclear extract is shown in Figure 1A. Jasp treatment decreased E‐cadherin, but increased N‐cadherin and vimentin, all of which are markers of EMT. LatB and CytD treatments increased E‐cadherin, but decreased N‐cadherin and vimentin levels (Figure 1F; Figure S1C, Supporting Information). Jasp treatment enhanced β‐catenin, SMAD2, and SMAD3, repressed MYBBP1A, NKRF, and MYPOP in the nuclei; while LatB and CytD enhanced MYBBP1A, NKRF, and MYPOP, repressed β‐catenin, SMAD2, and SMAD3 levels in the nuclei (Figure S1D, Supporting Information). Total actin input is shown in Figure S1D (Supporting Information) using GAPDH as a loading control for lysate, and PCNA as a loading control for nuclear extract.

### 2.2 MYBBP1A, NKRF, and MYPOP Suppress EMT

**Figure 2:**
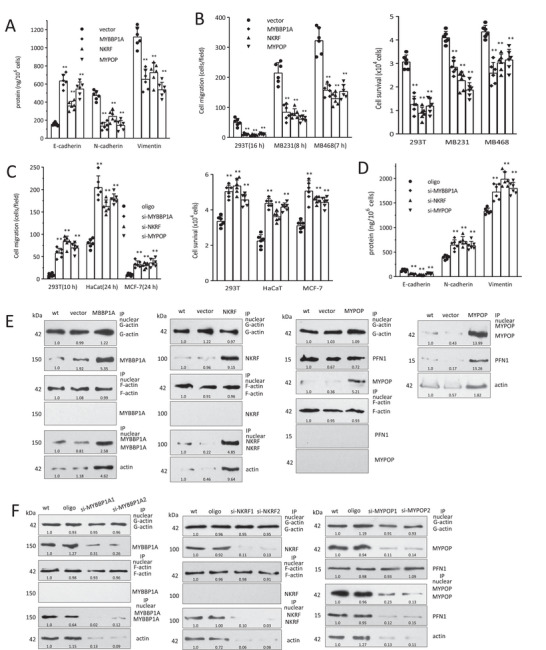
F/G‐actin binds EMT‐related functional transcription factors in the nuclei. A) HEK 293T cells were transfected with MYBBP1A, NKRF, or MYPOP, and processed to ELISA. Overexpression of MYBBP1A, NKRF, or MYPOP increased E‐cadherin, and decreased N‐cadherin and vimentin levels. **p < 0.01 versus vector (*n* = 6). B) Left, HEK 293T, MDA‐MB231, and MDA‐MB468 cells were transfected with MYBBP1A, NKRF, or MYPOP, and processed to chamber migration assays for indicated time points, showing that expression of MYBBP1A, NKRF, or MYPOP suppressed cell migration. **p < 0.01 versus vector (*n* = 6). Right, The transfected cells were cultured in basal medium with 700 µm H_2_O_2_ for 24 h. Expression of MYBBP1A, NKRF, or MYPOP suppressed cell survival. **p < 0.01 versus vector (*n* = 6). C) Left, HEK 293T, HaCaT, and MCF‐7 cells were transfected with MYBBP1A, NKRF, or MYPOP siRNAs, and processed to chamber migration assays for indicated time points, showing that silencing MYBBP1A, NKRF, or MYPOP enhanced cell migration. **p < 0.01 versus oligo (*n* = 6). Right, The cells were cultured in basal medium with 650 µm H_2_O_2_ for 24 h, showing that silencing MYBBP1A, NKRF, or MYPOP enhanced cell survival. **p < 0.01 versus oligo (*n* = 6). D) The transfected cell lysates were subjected to ELISA analysis, showing that silencing MYBBP1A, NKRF or MYPOP repressed E‐cadherin, and increased N‐cadherin and vimentin expression. **p < 0.01 versus oligo (*n* = 6). E) The nuclear extracts of the above transfected cells were lysed with LAS2, and subjected to F/G‐actin fractionation and immunoprecipitation with antibody against actin. Western blot showed that precipitation of nuclear G‐actin pulled down MYBBP1A, NKRF, and MYPOP in the nuclei, but precipitation of F‐actin did not pull down these proteins. Western blot showed that precipitation of MYBBP1A, NKRF, or MYPOP pulled down actin in the nuclei. F) Western blot showed that precipitation of nuclear G‐actin pulled down MYBBP1A, NKRF, and MYPOP, which pulled down less MYBBP1A (25.5%), NKRF (12.8%), and MYPOP (12.1%) in the siRNA knock‐down samples, but precipitation of F‐actin did not pull down MYBBP1A, NKRF, and MYPOP in the nuclear fraction. Precipitation of nuclear MYBBP1A, NKRF, and MYPOP also pulled down actin.

While there is evidence supporting the roles of β‐catenin, SMAD2, and SMAD3 in EMT, the functions of G‐actin binding transcription factors MYBBP1A, NKRF, and MYPOP in EMT are unknown. We found that MYBBP1A, NKRF, and MYPOP were highly expressed in HaCat and MCF‐7 cells, whereas they were expressed at low levels in MDA‐MB‐231 and MDA‐MB‐468 cells, and moderate levels in HEK‐293T cells (Figure S2A, Supporting Information). Overexpression of MYBBP1A, NKRF, and MYPOP yielded increased E‐cadherin, and repressed N‐cadherin and vimentin as tested by ELISA (**Figure** **2**A), RT‐PCR (Figure S2B, Supporting Information), Western blot (Figure S2C, Supporting Information), and immunostaining (Figure S2D, Supporting Information). MYBBP1A, NKRF, and MYPOP‐expressing cells showed suppressed cell migration, survival, and acquired cuboidal epithelial morphology (Figure 2B, Figure S3A,B, Supporting Information). Silencing MYBBP1A, NKRF, and MYPOP promoted cell elongation (Figure S3C, Supporting Information), and increased cell migration and survival (Figure 2C; Figure S4A, Supporting Information). Silencing MYBBP1A, NKRF, and MYPOP decreased E‐cadherin but increased N‐cadherin and vimentin expression (Figure 2D; Figure S4B,C, Supporting Information). Immunoprecipitation revealed the interaction of G‐actin with MYBBP1A, NKRF, and MYPOP in the nuclei (Figure 2E and Figure S4D, Supporting Information) and confirmed the binding of nuclear G‐actin with MYBBP1A, NKRF, and MYPOP (Figure 2F).

### 2.3 Nuclear F/G‐Actin Regulates EMT via Its Binding Transcription Factors

**Figure 3:**
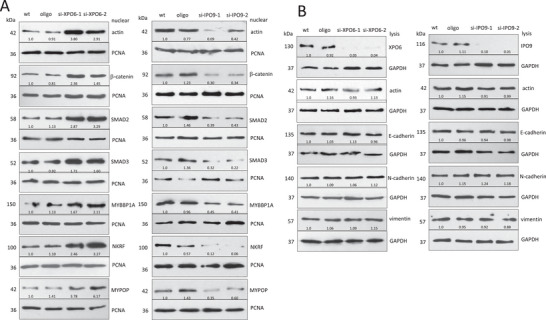
Silencing XPO6/IPO9 alters both F‐actin and G‐actin levels in the nuclei. A) HEK 293T cells were transfected with Exportin 6 (XPO6) or Importin 9 (IPO9) siRNAs, and subjected to nuclear fractionation. Western blot showed that silencing XPO6 with siRNAs (si‐XPO6) increased actin, β‐catenin, SMAD2, SMAD3, MYBBP1A, NKRF, and MYPOP in the nuclei, while silencing IPO9 (si‐IPO9) decreased actin, β‐catenin, SMAD2, SMAD3, MYBBP1A, NKRF, and MYPOP in the nuclei. B) Western blot showed that silencing XPO6/ IPO9 did not affect total actin, E‐cadherin, N‐cadherin, and vimentin expression levels in the cells.

Our gain‐and‐loss models identified MYBBP1A, NKRF, and MYPOP as EMT suppressors that showed the relevance of nuclear actin polymerization in regulating EMT via nuclear actin‐binding proteins: nuclear F‐actin promoted EMT through β‐catenin, SMAD2, and SMAD3, while nuclear G‐actin suppressed EMT via binding MYBBP1A, NKRF, and MYPOP. To validate these results, we designed cell models to specifically express different levels of nuclear F‐ or G‐actin with similar cell actin dynamics. Silencing nuclear export/import factor (Exportin 6/Importin 9, XPO6/IPO9) was an ideal approach to modulate nuclear F/G‐actin levels without altering cellular actin dynamics.^[^ ^39^ ^]^ We showed that silencing XPO6 increased actin, β‐catenin, SMAD2, SMAD3, MYBBP1A, NKRF, and MYPOP in the nuclei, while silencing IPO9 decreased the expression of these molecules in the nuclei (**Figure** **3**A). Silencing XPO6/IPO9 did not affect total cellular actin, E‐cadherin, N‐cadherin, and vimentin levels in the cells (Figure 3B).

**Figure 4:**
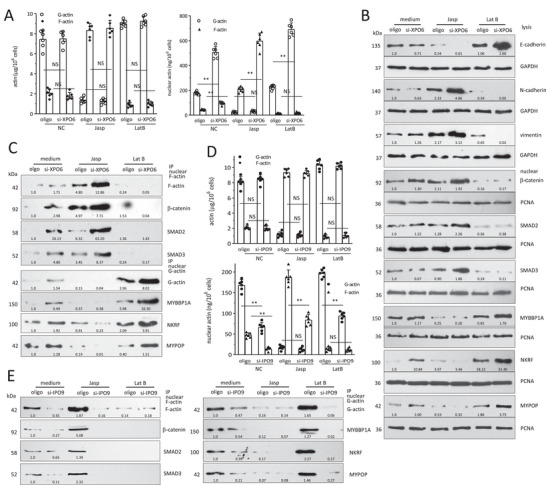
Effect of silencing XPO6/IPO9 on levels of nuclear F/G‐actin in the actin polymerization/depolymerization models. A) ELISA showed that medium/si‐XPO6 increased both F‐actin and G‐actin in the nuclei compared to the medium/oligo cells. Jasp/si‐XPO6 cells showed same cell actin dynamics and nuclear G‐actin levels as Jasp/oligo, and increased F‐actin in the nuclei. LatB/si‐XPO6 cells showed the same cell actin dynamics and nuclear F‐actin levels as LatB/oligo, and increased G‐actin in the nuclei. **p < 0.01 versus oligo (*n* = 6). B) The cells were lysed and subjected to Western blotting. Jasp/si‐XPO6 cells showed decreased E‐cadherin, but increased N‐cadherin and vimentin compared to Jasp/oligo, while LatB/si‐XPO6 cells presented increased E‐cadherin, but decreased N‐cadherin and vimentin compared to the LatB/oligo cells. Jasp/si‐XPO6 cells showed enhanced β‐catenin, SMAD2, and SMAD3 expression in the nuclei, while LatB/si‐XPO6 showed increased MYBBP1A, NKRF, and MYPOP expression in the nuclei compared to LatB/oligo treated cells. C) Nuclear F‐actin/G‐actin fraction from the above cells was subjected to immunoprecipitation with antibody against actin. Precipitation of F‐actin pulled down β‐catenin, SMAD2, and SMAD3, which was more evident in Jasp/si‐XPO6 cells. Precipitation of G‐actin pulled down MYBBP1A, NKRF, and MYPOP, which was more evident in LatB/si‐XPO6 cells. D) ELISA confirmed a decrease in both F‐actin and G‐actin in the nuclei of the medium/si‐IPO9 cells compared to the medium/oligo cells. Jasp/si‐IPO9 cells showed similar actin dynamics and nuclear G‐actin levels as Jasp/oligo, but decrease in F‐actin in the nuclei. LatB/si‐IPO9 cells showed similar actin dynamics and nuclear F‐actin levels as LatB/oligo, but decrease in G‐actin in the nuclei. **p < 0.01 versus oligo (*n* = 6). E) Nuclear F‐actin/G‐actin fraction was subjected to immunoprecipitation with antibody against actin. Precipitation of F‐actin pulled down β‐catenin, SMAD2 and SMAD3, while precipitation of G‐actin pulled down MYBBP1A, NKRF, and MYPOP.

To date, there has been a lack of a suitable cell model capable of selectively modulating nuclear actin polymerization without inducing global changes in cell actin dynamics. In order to investigate the impact of simultaneously applying actin‐binding compounds and siRNAs targeting XPO6/IPO9 on nuclear actin dynamics, HEK293T cells were transfected with XPO6 siRNAs or the control oligo and cultured in the medium containing 0.1 µm Jasp or 0.1 µm LatB for 24 h. In the control group, silencing XPO6 increased both F‐actin and G‐actin in the nuclei. The combined treatment with Jasp (Jasp/si‐XPO6) resulted in an increase in F‐actin levels specifically within the nuclei, with no significant changes observed in overall actin dynamics within the whole cell lysate when compared to the Jasp/oligo treatment group. On the other hand, the LatB/si‐XPO6 cells exhibited elevated levels of G‐actin within the nuclei, as demonstrated by ELISA (**Figure** **4**A) and Western blotting (Figure S5A, Supporting Information). The Jasp/si‐XPO6 cells showed that increased nuclear F‐actin repressed E‐cadherin, enhanced N‐cadherin and vimentin in the cells, and increased β‐catenin, SMAD2, and SMAD3 levels in the nuclei, while the LatB/si‐XPO6 cells displayed increased nuclear G‐actin, enhanced E‐cadherin, repressed N‐cadherin and vimentin in the cells, and increased MYBBP1A, NKRF and MYPOP expression in the nuclei (Figure 4B). Immunoprecipitation confirmed the interaction of nuclear F‐actin with β‐catenin, SMAD2, and SMAD3, and nuclear G‐actin with MYBBP1A, NKRF, and MYPOP (Figure 4C).

With a similar approach, the cells were transfected with IPO9 siRNAs or a control oligo and cultured in the presence of actin‐binding compounds. In the control group, silencing IPO9 decreased both F‐actin and G‐actin in the nuclei, and no significant actin dynamic change in the whole cell lysate compared with Jasp/oligo cells. Combined with Jasp treatment, the Jasp/si‐IPO9 cells showed decreased F‐actin in the nuclei, while the LatB/si‐IPO9 cells showed decreased G‐actin in the nuclei tested by ELISA (Figure 4D) and Western blot (Figure S5B, Supporting Information). The Jasp/si‐IPO9 cells showed decreased nuclear F‐actin, enhanced E‐cadherin, repressed N‐cadherin and vimentin in the cells, and decreased β‐catenin, SMAD2, and SMAD3 in the nuclei. The LatB/si‐IPO9 cells presented decreased nuclear G‐actin, repressed E‐cadherin, enhanced N‐cadherin and vimentin in the cells, and decreased MYBBP1A, NKRF, and MYPOP in the nuclei (Figure S5C, Supporting Information). Immunoprecipitation confirmed the interaction of nuclear F‐actin with β‐catenin, SMAD2 and SMAD3, and nuclear G‐actin with MYBBP1A, NKRF, and MYPOP in the nuclei (Figure 4E). These results showed that the combined application of actin‐binding compounds with siRNAs against XPO6/IPO9 was an ideal approach to study nuclear actin dynamics.

**Figure 5:**
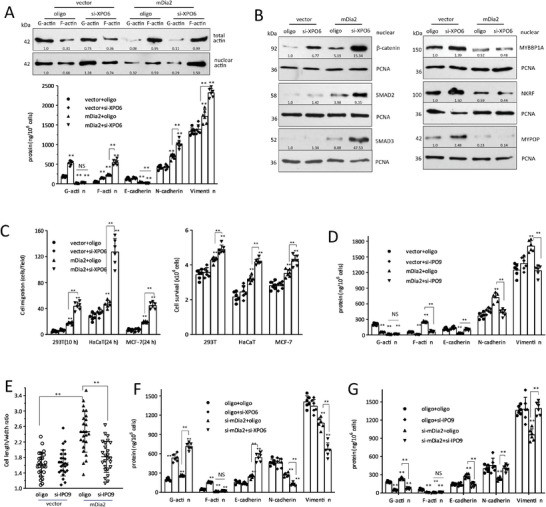
Nuclear F/G‐actin regulates EMT via binding and modulating β‐catenin, SMAD2, SMAD3, MYBBP1A, NKRF, and MYPOP expression in the nuclei. A) Upper, HEK293T cells were transfected with mDia2 with or without XPO6 siRNAs. The cells and nuclear extracts were subjected to F/G‐actin fractionation. Overexpression of mDia2 enhanced actin polymerization, and silencing XPO6 did not affect F/G‐actin in the cells. mDia2+/si‐XPO6 cells showed increased nuclear F‐actin. Lower, ELISA confirmed that mDia2+/si‐XPO6 cells expressed increased nuclear F‐actin, decreased E‐cadherin, but increased N‐cadherin and vimentin compared to mDia2+/oligo cells. **p < 0.01 versus oligo (*n* = 6). B) mDia2+/si‐XPO6 cells expressed increased β‐catenin, SMAD2, and SMAD3 in the nuclei compared to mDia2+/oligo cells. C) Left, HEK293T, HaCaT, and MCF‐7 cells were co‐transfected with mDia2 and XPO6 siRNAs, and processed to chamber migration assays for indicated time points. mDia2+/si‐XPO6 cells showed enhanced cell migration compared to mDia2+/oligo cells. Right, The transfected cells were cultured in basal medium with 650 µm H_2_O_2_ for 24 h. mDia2+/si‐XPO6 cells showed enhanced cell survival compared to mDia2+/oligo cells. **p < 0.01 versus oligo (*n = 6*). D) HEK293T cells were transfected with the control vector or mDia2 with or without IPO9 siRNAs. ELISA showed that mDia2+/si‐IPO9 cells expressed decreased nuclear F‐actin, increased E‐cadherin, but decreased N‐cadherin and vimentin compared to the mDia2+/oligo treated cells. **p < 0.01 versus oligo (*n* = 6). E) mDia2+/si‐IPO9 cells displayed cuboidal epithelial shape compared to mDia2+/oligo cells. The ratios of cell length/width were quantified, which showed that silencing IPO9 decreased cell elongation **p < 0.01 versus oligo (*n* = 25). F) HEK293T cells were transfected with mDia2 siRNAs with or without XPO6 siRNAs and processed for nuclear extraction. The cells and nuclear extracts were subjected to F/G‐actin fractionation. ELISA showed that si‐mDia2/si‐XPO6 cells expressed increased nuclear G‐actin, increased E‐cadherin, but decreased N‐cadherin and vimentin compared to mDia2+/oligo cells. **p < 0.01 versus oligo (*n* = 6). G) ELISA showed that si‐mDia2/si‐IPO9 cells expressed decreased nuclear G‐actin, decreased E‐cadherin, but increased N‐cadherin and vimentin expression compared to mDia2+/oligo treated cells. **p < 0.01 versus oligo (*n* = 6).

The commonly used actin binding compounds are shown to promote cell apoptosis by activating Caspase‐3 or other apoptotic pathways.^[^ ^40^, ^41^, ^42^ ^]^ To examine the effects of nuclear actin on EMT, we selected mDia2 as an actin filament stabilizer as reported.^[^ ^14^, ^43^ ^]^ Expression of mDia2 induced actin polymerization in both the nuclei and the cytosol, repressed cell E‐cadherin, and enhanced N‐cadherin and vimentin expression (Figure S5D,E, Supporting Information). Co‐transfection of mDia2 with XPO6 siRNA (mDia2+/si‐XPO6) increased nuclear F‐actin levels (**Figure** **5**A). mDia2+/si‐XPO6 cells showed decreased cell E‐cadherin, and increased N‐cadherin and vimentin (Figure S6A, Supporting Information), with increased β‐catenin, SMAD2 and SMAD3 in the nuclei (Figure 5B). The mDia2+/si‐XPO6 cells showed enhanced cell migration and survival, and displayed elongated mesenchymal morphology compared to mDia2+/oligo (Figure 5C, Figure S6B,C, Supporting Information). mDia2+/si‐IPO9 cells showed decreased nuclear F‐actin (Figure 5D and Figure S7A, Supporting Information). mDia2+/si‐IPO9 cells showed increased cell E‐cadherin, and decreased N‐cadherin and vimentin, with decreased β‐catenin, SMAD2, and SMAD3 in the nuclei (Figure S7B,C, Supporting Information). Functionally, mDia2+/si‐IPO9 cells showed repressed cell migration and survival, and displayed cuboidal epithelial shape compared to mDia2+/oligo treated cells. (Figure 5E; Figures S7D,F and S8, Supporting Information).

Silencing mDia2 induced actin depolymerization in the nuclei and the cytoplasm, which also increased cell E‐cadherin, and decreased N‐cadherin and vimentin expression (Figure S9A,B, Supporting Information). The si‐mDia2/si‐XPO6 cells showed increased nuclear G‐actin, increased MYBBP1A, NKRF, and MYPOP in the nuclei, and increased cell E‐cadherin, but decreased N‐cadherin and vimentin levels compared to si‐mDia2/oligo cells (Figure 5F; Figure S9C–E, Supporting Information). Functionally, si‐mDia2/si‐XPO6 cells showed decreased cell migration and survival, and loss of elongated mesenchymal morphology (Figure S10, Supporting Information), while si‐mDia2/si‐IPO9 cells expressed decreased nuclear G‐actin (Figure 5G and Figure S11A). The si‐mDia2/si‐IPO9 cells showed decreased MYBBP1A, NKRF, and MYPOP in the nuclei, and decreased cell E‐cadherin, while increased N‐cadherin and vimentin levels (Figure 5G; Figure S11B,C, Supporting Information). Functionally, si‐mDia2/si‐IPO9 cells showed enhanced cell migration and survival, and displayed elongated mesenchymal morphology compared to the si‐mDia2/oligo cells (Figure S12, Supporting Information). These experiments demonstrated that nuclear F‐actin promoted EMT by binding and increasing β‐catenin, SMAD2, and SMAD3, whereas, nuclear G‐actin suppressed EMT via binding and increasing MYBBP1A, NKRF, and MYPOP in the nuclei.

### 2.4 Overexpression of Nuclear F/G‐Actin Modulates EMT

**Figure 6:**
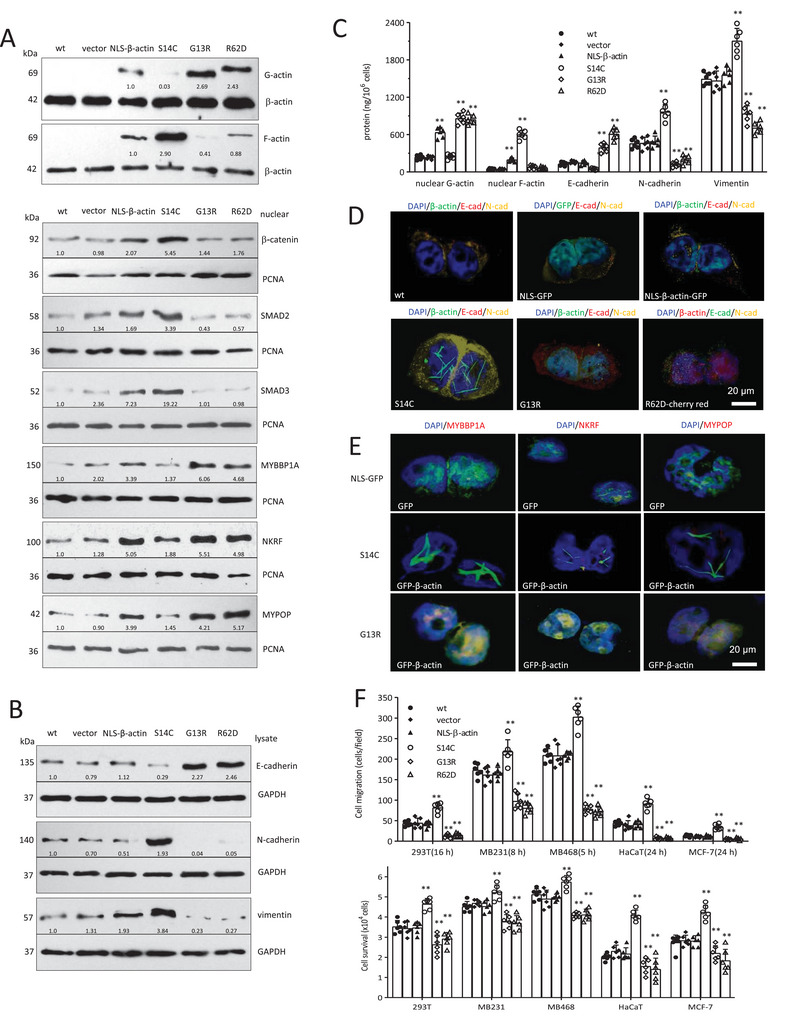
Overexpression of the nuclear F/G‐actin with constructs modulates EMT. A) Upper, HEK293T cells were transfected with YFP‐NLS‐β‐actin (NLS‐β‐actin), YFP‐NLS‐β‐actin S14C (S14C), YFP‐NLS‐β‐actin G13R (G13R), pmCherry‐NLS‐β‐actin R62D (R62D), and the vector. Transfection of NLS‐β‐actin expressed both F‐actin and G‐actin (β‐actin) in the nuclei, transfection of S14C expressed F‐actin (β‐actin), while transfection of G13R or R62D expressed G‐actin (β‐actin). Lower, The nuclear extracts were subjected to Western blotting. Transfection with S14C enhanced β‐catenin, SMAD2, and SMAD3, while transfection with G13R or R62D enhanced MYBBP1A, NKRF, and MYPOP levels in the nuclei. B) Transfection with S14C repressed E‐cadherin, and enhanced N‐cadherin and vimentin expression, while expression of G13R or R62D enhanced E‐cadherin, and repressed N‐cadherin and vimentin. C) ELISA showed that transfection with S14C repressed E‐cadherin, and enhanced N‐cadherin and vimentin expression, while expression of G13R or R62D enhanced E‐cadherin, and repressed N‐cadherin and vimentin. **p < 0.01 versus vector (*n* = 6). D) HEK293T cells were transfected with NLS‐β‐actin, S14C, G13R, or R62D. Immunofluorescence showed that transfection with S14C enhanced nuclear F‐actin and cell N‐cadherin levels, but repressed E‐cadherin levels. Transfection with G13R or R62D enhanced nuclear G‐actin and cellular E‐cadherin levels, but repressed N‐cadherin levels. E) HEK293T cells were transfected with S14C and G13R. Immunofluorescence showed the co‐localization of MYBBP1A, NKRF, and MYPOP with the nuclear G‐actin in the G13R‐transfected cells. F) Upper, HEK293T, MDA‐MB‐231, MDA‐MB‐468, HaCaT, and MCF‐7 cells were transfected with the above constructs, and processed to chamber migration assays for indicated time points. Transfection with actin S14C enhanced cell migration, while transfection with G13R or R62D repressed cell migration. Lower, The cells were cultured in basal medium with 700 µm H_2_O_2_ for 24 h. Transfection with S14C enhanced cell survival, while transfection with G13R or R62D repressed cell survival. **p < 0.01 versus vector (*n* = 6).

To validate the function of nuclear actin, we expressed YFP‐NLS‐β‐actin (NLS‐β‐actin, encoding a yellow fluorescent protein, or YFP, and nuclear localization signal, or NLS, tagged β‐actin), YFP‐NLS‐β‐actin S14C (or S14C, encoding YFP and NLS‐tagged β‐actin with the S14C polymerization mutation), YFP‐NLS‐β‐actin G13R (or G13R, encoding YFP and NLS‐tagged β‐actin with the G13R depolymerization mutation), and mCherry‐NLS‐β‐actin R62D (or R62D, expressing nuclear‐targeted non‐polymerizing R62D mutant of human actin, with an mCherry expression reporter) in HEK293T cells. Transfection of NLS‐β‐actin increased F/G‐actin, β‐catenin, SMAD2, SMAD3, MYBBP1A, NKRF, and MYPOP in the nuclei; S14C increased F‐actin, β‐catenin, SMAD2, and SMAD3 in the nuclei; G13R or R62D increased G‐actin, MYBBP1A, NKRF and MYPOP in the nuclei (**Figure** **6**A). NLS‐β‐actin did not affect cell E‐cadherin, N‐cadherin. and vimentin levels; S14C decreased E‐cadherin, increased N‐cadherin and vimentin; G13R and R62D increased E‐cadherin, decreased N‐cadherin and vimentin (Figure 6B–D; Figure S13, Supporting Information: profiles of individual staining in Figure 6D are provided in Figure S13A, Supporting Information). Immunoprecipitation confirmed the interaction of nuclear F‐actin with β‐catenin, SMAD2, and SMAD3, and nuclear G‐actin with MYBBP1A, NKRF, and MYPOP, with high levels in G13R and R62D cells (Figure S14, Supporting Information). Immunofluorescence staining showed co‐localization of MYBBP1A, NKRF, and MYPOP with nuclear G‐actin in G13R and R62D cells (Figure 6E, profiles of individual staining provided in Figures S15 and 16, Supporting Information). Functionally, S14C cells displayed enhanced migration and survival, and elongated mesenchymal shape; G13R and R62D cells exhibited repressed migration and survival, and induced cuboidal epithelial structure (Figure 6F; Figure S17, Supporting Information).

### 2.5 Nuclear Actin Polymerization Regulates EMT by Modulating β‐Catenin, SMAD2, SMAD3, MYBBP1A, NKRF, and MYPOP Stability in the Nuclei

**Figure 7:**
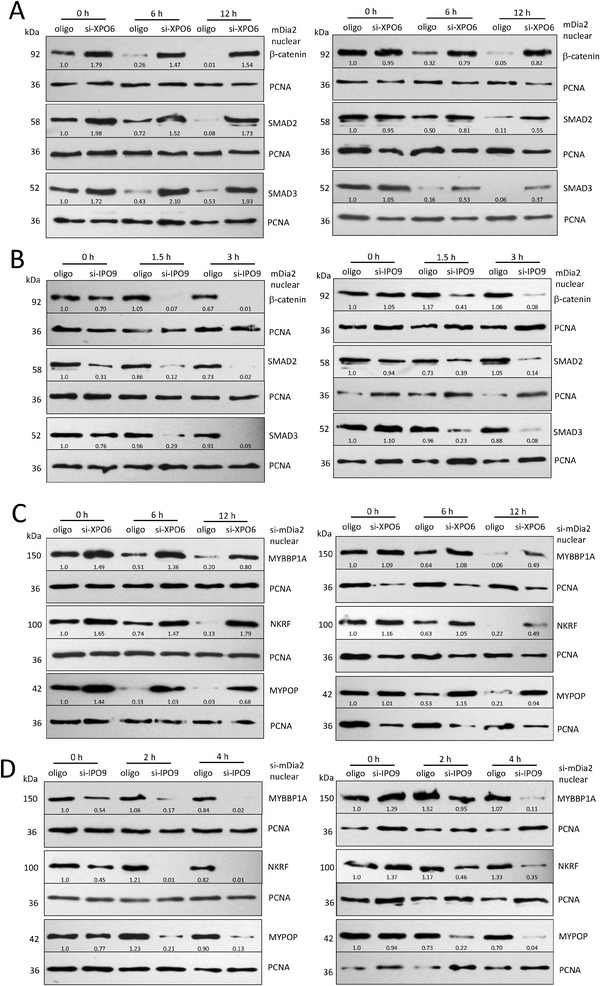
Nuclear actin polymerization regulates EMT via modulating β‐catenin, SMAD2, SMAD3, MYBBP1A, NKRF, and MYPOP stability in the nuclei. A) Left, HEK293T cells were co‐transfected with mDia2 and XPO6 siRNAs and cultured in 20 µm Emetine for indicated time points. The nuclear extracts were lysed and subjected to Western blotting. mDia/si‐XPO6 cells showed enhanced β‐catenin, SMAD2, and SMAD3 expression in the nuclei compared to mDia/oligo cells. Right, The amounts of loaded samples were adjusted to contain the same protein expression levels at the starting point. β‐catenin, SMAD2, and SMAD3 showed increased stability in mDia/si‐XPO6 nuclei compared to mDia/oligo cells. B) Left, HEK293T cells were co‐transfected with mDia2 and IPO9 siRNAs, and cultured in 20 µm Emetine. mDia/si‐IPO9 cells showed decreased β‐catenin, SMAD2, and SMAD3 expression in the nuclei compared to mDia/oligo cells. Right, The amounts of samples loaded were adjusted to contain the same protein expression levels at the starting point. β‐catenin, SMAD2, and SMAD3 showed decreased stability in mDia/si‐IPO9 nuclei relative to mDia/oligo. C) Left, HEK293T cells were co‐transfected with mDia2 siRNAs and XPO6 siRNAs, and cultured in 20 µm Emetine. si‐mDia/si‐XPO6 cells showed enhanced MYBBP1A, NKRF, and MYPOP expression in the nuclei compared to si‐mDia/oligo cells. Right, The amounts of samples loaded were adjusted to contain the same protein expression levels at the starting point. MYBBP1A, NKRF, and MYPOP showed increased stability in si‐mDia/si‐XPO6 cell nuclei compared to si‐mDia/oligo cells. D) Left, HEK293T cells were co‐transfected with mDia2 siRNAs and IPO9 siRNAs, and cultured in 20 µm Emetine. The si‐mDia/si‐IPO9 cells expressed decreased MYBBP1A, NKRF, and MYPOP in the nuclei compared to the si‐mDia/oligo cells. Right, MYBBP1A, NKRF, and MYPOP showed decreased stability in the si‐mDia/si‐IPO9 cell nuclei compared to the si‐mDia/oligo cells.

To study how nuclear actin regulated EMT‐related transcription factors, mDia2+/si‐XPO6 cells were cultured in 20 µm Emetine. These cells expressed higher nuclear levels of β‐catenin, SMAD2, and SMAD3 than mDia2+/oligo cells (**Figure** **7**A, left). The stability of nuclear β‐catenin, SMAD2, and SMAD3 was compared by modifying loaded quantities (having same protein expression levels at the starting point). In this case, mDia2+/si‐XPO6 showed higher levels of the above proteins at 6 and 12 h, indicating that these proteins displayed enhanced stability compared to mDia2+/oligo (Figure 7A, right). mDia2+/si‐IPO9 cells were also cultured in 20 µm Emetine, which showed decreased stability of β‐catenin, SMAD2, and SMAD3 compared to mDia2+/oligo cells (Figure 7B). Using a similar approach, we treated mDia2‐/si‐XPO6 and mDia2‐/si‐IPO9 cells with Emetine, and observed that expression of nuclear G‐actin increased MYBBP1A, NKRF, and MYPOP stability, whereas decreased nuclear G‐actin levels decreased MYBBP1A, NKRF, and MYPOP stability in the nuclei (Figure 7C‐D). Expression of nuclear F‐actin enhanced β‐catenin, SMAD2, and SMAD3 stability, while expression of nuclear G‐actin increased MYBBP1A, NKRF, and MYPOP stability in the nuclei. Thus, nuclear actin dynamics regulate EMT by modulating the expression of actin‐binding transcription factors.

### 2.6 Correlation of Nuclear Actin Polymerization and EMT in Cell Lines and Skin

**Figure 8:**
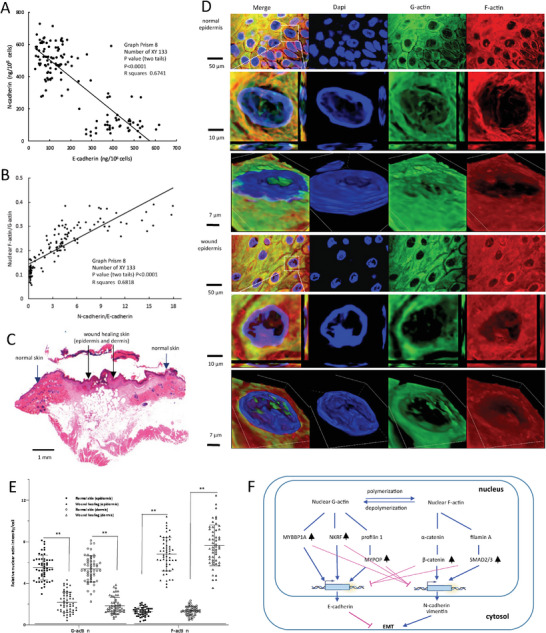
Association of EMT with wound repair. A) Absolute values of E‐cadherin, N‐cadherin, vimentin, and nuclear F‐actin/G‐actin protein levels were quantified by ELISA in 133 cell lines including, MDA‐231‐BoM‐1833, 786‐O, 4T1, 4T07, 66C14, 67NR, A431, A549, A2058, A2780, AC16, ACHN, ARH77, AU565, B16, BC3, BEAS‐2B, BTH‐1, BT‐20, BT‐474, BT‐549, BxPC‐3, C2C12, Caco‐2, Caki‐1, Caki‐2, CI3K, CO‐115, COLO‐201, COLO‐205, COLO‐775, CW‐9019, CRL‐1476, Cos‐1, Cos‐7, CV‐1, DLD‐1, DU145, EMT6, ES2, FHC, H460, HaCaT, HCC1393, HCT6, HCT8, HCT15, HCT116, HDL100, HEK293T, Hela, Hep3B, HepG2, HEY, HGF, HL‐1, HL‐60, HLE, HT‐1080, HT‐29, HTB‐123, HTB‐126, Ho, Hs5787, Huh6, Huh7, ICE6, ICE18, JHH‐1, Jurkat, JR75‐1, JR‐75‐30, K652, KTC‐1, LAPC‐4, LAPC‐9, Li7, LNCaP, MC3T3, MCF, MCF‐7, MCF‐10A, MDA‐MB‐157, MDA‐MB‐175, MDA‐MB‐231, MDA‐MB‐436, MDA‐MB‐468, MDA‐MB‐453, NIH3T3, NMuMG, PAN3, PANC‐1, PC3, PC12, PLC/PRF/5, OV‐2008, OVCAR‐3, Raji, Rat2, RD, RFL‐6, RH1, RH2, RH3, RH4, RH6, RH14, RH18, RH28, RH30, RIE‐1, SK‐NEP‐1, SNU‐16, SNU‐378, SNU‐449, Saos‐2, SW48, SW480, SW620, SW837, SW1116, SW1353, T3M‐4, T47V, T860, TOV‐112, SK‐BR‐3, UO‐31, U87, U118, U343, U937, and YPEN‐1. Pearson correlation analysis showed that E‐cadherin levels were negatively correlated with N‐cadherin in the cell lines. *p* < 0.0001, *n* = 133, *R* ^2^ = 0.6741. B) Pearson correlation analysis showed a positive correlation between the ratio of nuclear F‐actin/G‐actin proteins and N‐cadherin/E‐cadherin. *p* < 0.0001, *n* = 133, *R* ^2^ = 0.6818. C) A total of 52 wound healing samples were selected, which were collected from 6 day‐wounded mice. All samples contained normal skin and wound healing skin in the same section, which was confirmed by H&E staining. A typical image of wound healing sample is shown. D) Typical z‐stack images (xy, xz, and yz projection and orthogonal view) showing nuclear F‐actin (Phalloidin staining, red) and G‐actin (Deoxyribonuclease I staining, green) in wound healing and normal skin cells (epidermis). The images for wound healing skins were randomly selected from central wound healing areas, while the normal skin images were randomly selected from the normal epidermis areas far from the wound region. E) ImageJ analysis showed that the wound healing cells expressed higher levels of nuclear F‐actin and lower levels of nuclear G‐actin than the normal skin cells. The intensity of Phalloidin (F‐actin)/Deoxyribonuclease I staining (G‐actin) within the cell nucleus (DAPI staining) was analyzed by ImageJ. Phalloidin/Deoxyribonuclease I staining regions that overlapped with DAPI were defined as nuclear F/G‐actin stained. The average intensity value of five cells from each image represented F/G‐actin intensity of the sample image. Phalloidin/Deoxyribonuclease I stained areas around the edge of the nucleus were excluded, and only the regions stained away from the nuclear edge were counted as stained positive for nuclear F/G‐actin. **p < 0.01 versus normal (*n* = 6). F) A diagram showing that nuclear actin polymerization regulates cell epithelial‐mesenchymal transition process.

To evaluate the correlation of nuclear actin dynamics with EMT process in the cell lines, absolute values of nuclear F/G‐actin proteins and EMT markers were measured by ELISA in 133 cell lines. Pearson correlation analysis showed negative correlation between E‐cadherin and N‐cadherin in these cell lines (**Figure** **8**A). The ratio of nuclear F‐actin/G‐actin was negatively correlated with E‐cadherin, while it was observed to be positively correlated with N‐cadherin, vimentin, and N‐cadherin/E‐cadherin (Figure 8B; Figure S18, Supporting Information).

EMT plays key roles in skin wound healing processes.^[^ ^44^, ^45^ ^]^ To observe nuclear actin dynamics in this process, 52 mouse wound healing samples were obtained from 6‐day‐wounded mice, containing normal as well as wounded skin (Figure 8C). Staining analysis showed that cells in the epidermis (Figure 8D) and dermis (Figure S19A, Supporting Information) of the wound expressed higher levels of nuclear F‐actin but lower levels of nuclear G‐actin than the normal skin cells, indicating the role of nuclear actin polymerization in the EMT associated wound healing process. Quantitation analysis confirmed that both epidermis and dermis of the wound tissues expressed higher levels of nuclear F‐actin but lower levels of nuclear G‐actin than normal skin (Figure 8E). Increase in nuclear F‐actin and decrease in nuclear G‐actin contributes to EMT by regulating the related signaling pathways (Figure 8F). Nevertheless, there was no significant difference between total F‐actin and G‐actin (Figure S19B, Supporting Information).

## 3 Discussion

Nuclear translocation of β‐catenin, SMAD2, and SMAD3 has been reported to play roles in the activation of these EMT‐related transcription factors.^[^ ^29^, ^46^ ^]^ The binding of β‐catenin to nuclear F‐actin via interaction with α‐catenin increases β‐catenin stability in the nucleus,^[^ ^25^, ^26^ ^]^ which is essential in the regulation of Wnt/β‐catenin signaling and EMT.^[^ ^22^, ^28^, ^29^ ^]^ FLNA, an F‐actin binding partner has been reported to interact with SMAD2 and SMAD3.^[^ ^32^, ^47^ ^]^ Activation of SMADs results in their translocation from cytoplasm into nucleus, and promotes cellular EMT.^[^ ^48^ ^]^ Interestingly, our study indicates that nuclear F‐actin binds β‐catenin, SMAD2 and SMAD3, and enhances these EMT‐enhancing transcription factors’ stability in the nuclei.

To our surprise, we found that nuclear G‐actin could bind MYBBP1A, NKRF, and MYPOP and enhanced these EMT‐suppressing transcription factors’ stability in the nuclei. MYBBP1A has been described as a nucleolar protein, which acts as a co‐repressor of multiple transcription factors involved in various physiological processes.^[^ ^35^ ^]^ MYBBP1A functions as a tumor suppressor by regulating c‐MYB and PGC1α.^[^ ^49^ ^]^ Loss of MYBBP1A induces cancer stem cell activity, metastasis, and EMT.^[^ ^36^ ^]^ NKRF, as a nucleolar protein, is essential for nucleolar homeostasis.^[^ ^50^ ^]^ It interacts with specific negative regulatory elements (NREs) and represses NF‐kappa‐B transcription.^[^ ^51^ ^]^ Down‐regulation of NKRF elevates NF‐kappa‐B activation and promotes tumor progression.^[^ ^52^ ^]^ The best‐known G‐actin binding protein PFN1, a regulator of the cytoplasmic actin dynamics was reported to bind to several nuclear proteins including MYPOP, thus regulating its activity.^[^ ^33^, ^34^, ^53^ ^]^ Interestingly, all of these three reported G‐actin binding proteins are transcription factors, mainly expressed in the nuclei, possessing tumor‐suppressing functions.^[^ ^35^, ^36^, ^38^ ^]^ Our results showed that nuclear G‐actin binds MYBBP1A, NKRF, and MYPOP, and increases these nucleolar proteins’ stability in the nuclei. Expression of MYBBP1A, NKRF, or MYPOP suppressed cell survival, migration, and EMT, whereas silencing MYBBP1A, NKRF, or MYPOP enhanced cell survival, migration, and EMT.

To observe how nuclear actin regulated EMT, we generated a cell model that specifically expressed different levels of the nuclear F/G‐actin with the same cellular actin dynamics. Combining actin filament stabilizer or inhibitor with the siRNAs against nuclear export/import factors for actin (XPO6/IPO9), we specifically expressed or silenced nuclear F/G‐actin and studied the nuclear actin function in comparison to the control samples with the same cell actin dynamics. This uncovered that nuclear F‐actin promoted cell EMT, with decreased E‐cadherin, and increased N‐cadherin and vimentin. Nuclear F‐actin enhanced cell migration and survival, and elongated mesenchymal morphology. Contrarily, the nuclear G‐actin repressed EMT with suppressed cell migration and survival, and induced formation of a cuboidal epithelial structure. Our results were further confirmed by expressing NLS–β‐actin and/or its mutant constructs which exogenously expressed F‐actin/G‐actin in the nucleus. A positive association between the nuclear F/G‐actin ratio and N‐cadherin/E‐cadherin was detected in the studied 133 cell lines. Increased nuclear actin polymerization was also observed in the typical EMT process of the skin wound healing cells, which provided in vivo evidence signifying the role of nuclear actin polymerization in EMT.

Our results revealed a notable role of nuclear actin polymerization in regulating the cell EMT process. The dynamic equilibrium between F‐actin and G‐actin, accurately regulated by polymerization and depolymerization, is crucial in controlling cell EMT. Actin binds functional transcription factors related to EMT in the nuclei, including F‐actin binding β‐catenin, SMAD2, and SMAD3, and G‐actin binding MYBBP1A, NKRF, and MYPOP. Expression of the nuclear F‐actin enhances β‐catenin, SMAD2, and SMAD3 stability in the nuclei, whereas nuclear G‐actin increases MYBBP1A, NKRF, and MYPOP stability in the nuclei. Activation of β‐catenin, SMAD2, and SMAD3 in the nuclei represses E‐cadherin, while enhancing N‐cadherin and vimentin transcription. Meanwhile, expression of MYBBP1A, NKRF, and MYPOP increases E‐cadherin, and decreases N‐cadherin and vimentin transcription. Our results confirmed the roles of β‐catenin, SMAD2, and SMAD3 in promoting EMT, and identified the roles of tumor suppressor MYBBP1A, NKRF, and MYPOP in repressing the EMT process. Hence, it can be deduced that nuclear actin exerts a role in the EMT process by dynamic polymerization and depolymerization of F‐actin and G‐actin that bind and regulate different signaling molecules and modulate transcription events in the nuclei.

## 4 Experimental Section

### Materials

Monoclonal antibodies against β‐catenin (#8480), SMAD2 (#5339), SMAD3 (#9523), E‐cadherin (#14472), N‐cadherin (#13116), vimentin (#5741), and actin (#4968) were purchased from Cell Signaling Technology (Danvers, MA, USA). Monoclonal antibodies against FLNA (A3738) and profilin‐1 (A1164), and polyclonal antibodies against Myb‐binding protein 1A (MYBBP1A, A4429), NF‐kappa‐B‐repressing factor (NKRF, A4853), E‐cadherin (A3044), N‐cadherin (A0432), and vimentin (A11952) were purchased from ABclonal (Woburn, MA, USA). Polyclonal antibodies against Myb‐related transcription factors (MYPOP, NBP2‐55767 and NBP2‐83249) were purchased from Novus Biologicals (Littleton, CO, USA). Monoclonal antibody against β‐actin (A5441), polyclonal antibody against α‐catenin (C2081), Alexa Fluor 488 Deoxyribonuclease I (D12371), Alexa Fluor 555 Phalloidin (A34055), Western blot detection kit (C72652), and 96‐well ELISA high binding plate (MSEHNFX) were purchased from MilliporeSigma (Oakville, ON, Canada). Human actin protein (APHL99), G‐actin/F‐actin in vivo assay kit (BK037) and monoclonal antibody against Actin (AAN02) were purchased from Cytoskeleton, Inc (Denver, CO, USA). Human E‐cadherin (10204), N‐cadherin (11039) and vimentin (10028) proteins, and polyclonal antibody against actin (101273) were purchased from Sino Biological. Horseradish peroxidase‐conjugated goat anti‐rabbit and anti‐mouse IgG, protein G magnetic beads (161‐4023), RNA RT (1725151) and PCR (1708880) kits were purchased from Bio‐Rad (Hercules, CA, USA). Biotin‐XX Phalloidin (B7474), Dynabeads^TM^ MyOne^TM^ Streptavidin C1(65002), and 8.0 µm pore size chamber migration kit (113819) were purchased from Thermo Fisher Scientific (Waltham, MA, USA).

### Constructs, siRNAs, and Primers

The plasmids containing full‐length human MYBBP1A, YFP‐NLS‐β‐actin, YFP‐NLS‐β‐actin S14C, YFP‐NLS‐β‐actin G13R, and pmCherry‐NLS‐β‐actin R62D were obtained from Addgene (Watertown, MA, USA). NKRF was from Creative Biogene (Shirley, NY, USA). MYPOP was from DNASU (Tempe, AZ, USA). All primers and siRNA sequences used in the study are listed in Table S2 (Supporting Information).

### Transient Transfection of Mammalian Cells

Cells were cultured in six‐well culture dishes containing basal medium supplemented with 10% FBS (5 × 10^5^ cells per well) and maintained at 37 °C for 16 h. After washed with PBS, the attached cells were cultured in serum‐free medium transfected with PolyJet (3 µL mL^−1^) and plasmids or siRNAs (2 µg mL^−1^) for 5 h, and maintained in 10% FBS basal medium for 24 h before RNA and protein analysis or passed to new culture plates for functional assays, including migration or survival tests.

### Cell Survival Assays

Cells were cultured in 10% FBS basal medium in 12‐well culture dishes (5 × 10^4^ cells per well), and maintained at 37 °C for 16 h. The cultured cells were replaced with 10% FBS basal medium containing H_2_O_2_ with indicated concentration for indicated time points. The harvested cells were stained with Trypan Blue, and cell number was counted by a Coulter Counter under the inverted microscope.

### Chamber Cell Migration Assays

A polyethylene terephthalate (PET) membrane cell culture insert (Falcon, 1138019, Thermo Fisher Scientific) was placed in 24‐well tissue culture plates, and 1×10^5^ cells in 200 µL media without fetal bovine serum (FBS) were loaded into the upper part of the chamber membrane. Each well was filled with 800 µL DMEM containing 10% FBS. After incubation at 37 °C for different time points, the non‐migratory cells were removed with a cotton swab, and the migratory cells were fixed with 100% methanol for 30 min, followed by staining with Coomassie blue for 5 min. Images were taken under an inverted light microscope for quantitation. All the migration assays were performed in the medium with 2 µg mL^−1^ mitomycin C.

### Immunofluorescence Staining

Cells cultured on BD culture slides were fixed for 20 min in 3.7% formaldehyde solution, blocked with 10% goat serum, followed by overnight incubation with primary antibody in PBS containing 10% goat serum. The slides were washed and incubated with Alexa Fluor 488, 555, or 647 secondary antibodies at room temperature for 2 h. DAPI was used to stain DNA. Images of the stained samples were taken using Nikon N‐SIM S confocal laser scanning microscopy. All confocal images shown are single‐plane views, except those mentioned in the figures. The intensity of staining was analyzed by ImageJ.

### Analysis of Nuclear F/G‐Actin in Wound Healing Samples

Total 52 wound healing samples were obtained in a skin wound healing model as described.^[^ ^54^ ^]^ The samples were collected from 6 day‐wounded mice. All samples contained normal skins and wound‐healing skins in the same section, which were confirmed by H&E staining. Briefly, sample sections were de‐paraffinized with xylene and ethanol, washed with Tris‐Buffered‐Saline (TBS) containing 0.025% Triton X‐100. The sections were blocked with 10% goat serum and incubated with 1:3000 Alexa Fluor 488 conjugated Deoxyribonuclease I (D12371) in TBS containing 10% goat serum at 4 °C overnight. After washed with TBS, the sections were incubated with 1:100 Alexa Fluor 555 Phalloidin (A34055) and DAPI for 30 min. The images for wound‐healing skin were randomly selected from the central wound healing area (epidermis and dermis), while the normal skin images were randomly selected from the normal epidermis and dermis region far from the wound area. Images of the stained samples were obtained using Nikon N‐SIM S confocal laser scanning microscopy. Five single cells were randomly selected from each image. The intensity of Phalloidin (F‐actin)/Deoxyribonuclease I staining (G‐actin) within the cell nucleus (DAPI staining) was analyzed by ImageJ. The regions of Phalloidin/Deoxyribonuclease I staining that overlapped with DAPI were defined as nuclear F/G‐actin stained. The average intensity value of five cells from each image represented F/G‐actin intensity of the sample image. However, Phalloidin/Deoxyribonuclease I staining around the edge of the nucleus was excluded, and only the staining area away from the nuclear edge was counted as nuclear F/G‐actin positive.

### Immunoprecipitation Assays

Bio‐Rad magnetic beads were used for immunoprecipitation assays. Briefly, 100 µL magnetic beads were washed in PBS‐T (PBS + 0.1% Tween 20) and incubated with 5 µg primary antibody at room temperature for 10 min. Cells or tissues were washed, lysed, and incubated with antibody‐containing beads for 1 h. The magnetic beads were washed 3 times with PBS‐T and resuspended in 2× Laemmli buffer (0.125 m Tris‐HCl, 4% SDS, 20% glycerol, 10% 2‐mercaptoethanol, 0.004% bromophenol blue, pH 6.8), followed by Western blot analysis. All the immunoprecipitation assays were repeated three times except described in the figure legends.

### Western Blotting

Cells or tissues were lysed and subjected to sodium dodecyl sulfate‐polyacrylamide gel electrophoresis (SDS‐PAGE) containing 7–12% acrylamide. Transblotting was performed onto a nitrocellulose membrane in 1× Tris/glycine buffer containing 20% methanol at 60 V at 4 °C for 2–4 h. The membrane was blocked in a buffer containing 10 mm Tris‐Cl, pH 8.0, 150 mm NaCl, 0.05% Tween‐20, and 5% non‐fat dry milk powder for 0.5 h, following incubation with primary antibodies at 4 °C overnight. The membranes were washed with the above washing buffer 3×20 min, and incubated with secondary antibodies for 2 h. After washing with washing buffer 3 × 20 min, the bound antibodies were visualized with an ECL detection kit. The intensities of protein bands were quantified by densitometry and labeled below the bands.

### Cell Nuclear Extraction

Cultured cells were harvested and resuspended in 500 µL fractionation buffer (250 mm sucrose, 20 mm HEPES pH 7.4, 10 mm KCl, 2 mm MgCl_2_, 1 mm EDTA, 1 mm EGTA, and 1× Roche protease inhibitor cocktail). After, the cells were homogenized by 10 passages through a 25‐G needle using a 2 mL syringe and incubated on ice for 30 min. To keep the stability of nuclear actin polymerization, cells were suspended in fractionation buffer and centrifuged at low speed (720 × *g*) at room temperature for 10 min. The pellet contained nuclear fraction. The nuclear pellet was resuspended in 500 µL fractionation buffer followed by centrifugation at 720 × *g* for 5 min. To maximally decrease contamination of nuclear fraction by cytoplasm, resuspension, and centrifugation were repeated three times. The purity of isolated nuclear and cytosolic fractions was checked by Western blot with antibodies against tubulin and PCNA. The extracted nuclear pellet was used for further experiments or kept frozen in −80 °C refrigerator.

### F/G‐Actin Fraction

F/G‐actin in vivo assay kit (BK037) was used to isolate F‐actin and G‐actin fractions. Briefly, the cultured cells or isolated nuclei were lysed with lysis buffer and F‐actin stabilization buffer 2 (LAS2), homogenized with 25 G syringe with a bent‐over tip 10 times, and incubated at 37 °C for 10 min. After centrifuged at 2000 rpm for 5 min to pellet the unbroken cells, the supernatant was centrifuged at 100 000 × *g* at 37°C for 1 h. After centrifugation, the pellet contained F‐actin and its binding proteins, while the supernatant contained G‐actin.

### Identification of G‐Actin Binding Proteins

Bio‐Rad magnetic beads were used for immunoprecipitation of G‐actin and its binding proteins. Briefly, 100 µL magnetic beads were washed in PBS‐T (PBS + 0.1% Tween 20) and incubated with 5 µg monoclonal antibody against actin at room temperature for 10 min. The actin antibody‐conjugated magnetic beads were washed with PBS‐T for three times. Meanwhile, the cultured cells or isolated nuclei were lysed with LAS2, and subjected to F/G‐actin fractionation. After centrifuging at 100 000 × *g* at 37 °C for 1 h, the supernatant containing G‐actin was incubated with actin antibody‐containing beads for 1 h. The magnetic beads were washed three times with PBS‐T and resuspended in 2× Laemmli buffer (0.125 m Tris‐HCl, 4% SDS, 20% glycerol, 10% 2‐mercaptoethanol, 0.004% bromophenol blue, pH 6.8), followed by Western blot analysis.

### Identification of F‐Actin Binding Proteins

Bio‐Rad magnetic beads were used for immunoprecipitation of F‐actin binding proteins. Briefly, the culture cells or isolated nuclei were lysed with LAS2, and subjected to F/G‐actin fractionation. After centrifuging at 100 000 × *g* at 37 °C for 1 h, the pellet containing F‐actin and its binding proteins was resuspended in 500 µL LAS2, and incubated with actin antibody‐containing beads for 1 h. The magnetic beads were washed three times with LAS2 and resuspended in 2× Laemmli buffer (0.125 m Tris‐HCl, 4% SDS, 20% glycerol, 10% 2‐mercaptoethanol, 0.004% bromophenol blue, pH 6.8), followed by Western blot analysis.

F‐actin and its binding proteins were also immunoprecipitated with Phalloidin. Briefly, the F‐actin and its binding proteins containing fraction was resuspended in 500 µL LAS2, and incubated with 25 µL biotin‐XX Phalloidin (B7474) at 37 °C for 30 min. 50 µL Dynabeads MyOne Streptavidin C1(65002) were washed, added to each binding reaction, and incubated at 37 °C for another 30 min. The beads were washed with LAS2 three times, and the binding proteins in the pull‐down products were analyzed by Western blotting.

### ELISA

Absolute values of F/G‐actin, E‐cadherin, N‐cadherin, and vimentin in the cells were quantified on the basis of standard protein curve in enzyme‐linked immunosorbent assay (ELISA). Briefly, 100 µL of polyclonal rabbit antibody against the above‐mentioned proteins (0.5 µg mL^−1^ diluted 1:1000 in 0.2 m carbonate/bicarbonate buffer, pH 9.6) was added to each well of a 96‐well ELISA high binding plate (MSEHNFX, Sigma), and incubated overnight at 4 °C. The plates were then washed with PBS, followed by blocking (5% non‐fat dry milk/PBS) for 1 h. After washing, 100 µL of each sample and the standardly purified proteins were loaded and incubated for 2 h at 37 °C. After washing, the mouse monoclonal antibodies against the above‐mentioned proteins (1:500–2000) were added and incubated for 2 h, followed by the addition of goat anti‐mouse secondary antibody (HRP, 1:4000) for 2 h. The plates were then washed and incubated with TMB Substrate Solution (Thermo Scientific) for 30 min. The reaction was stopped with 2 m H_2_SO_4_, followed by optical density measurement at 450 nm. Standard curves were drawn and OD values were then interpolated to determine the sample protein concentrations. Each sample was tested in duplicates. The absolute value of nuclear F/G‐actin in the cells was also evaluated using the same method.

### RT‐PCR and Real‐Time PCR

The tissues or cells were harvested, and total RNA was extracted with the RNA mini kit (Cat# GZXD200, Geneaid). Real‐time PCR was performed with SYBR Green PCR Kit (Cat# 1725120, Bio‐Rad) using 2 µL cDNA as a template with two gene‐specific primers. Thermocycler conditions were set as: 35 cycles of denaturation at 95 °C for 15 s, annealing at 56 °C for 10 s, and extension at 72 °C for 5 s. The ΔΔCT method was used to quantify all the relative mRNA levels using small nuclear RNA U6 as an internal control.

### Statistical Analysis

Data were presented as mean ± standard deviation (SD). Shapiro–Wilk normality test (*n* < 8) or D'Agostino and Pearson normality test (*n* ≥ 8) was used to check whether populations followed a Gaussian distribution. Bartlett's test was used when Gaussian distribution was present, whereas Brown–Forsythe test was used when the data were skewed. For multiple group analyses, one‐way ANOVA followed by a Bonferroni post hoc test for one independent variable, and two‐way ANOVA followed by Bonferroni correction for two independent variables were performed. Two‐tailed unpaired Student's *t*‐test was performed to assess the difference between the two groups with a single independent factor. When normal distribution was not confirmed, a non‐parametric two‐tailed unpaired Mann–Whitney or Kruskal‐Wallis test was performed, followed by Dunn's correction. All in vitro experiments were repeated at least three times, except otherwise described. Pearson correlation was used to analyze the linear relationship between two variables. Prism 8 (GraphPad Software: La Jolla, CA) was used for the above statistical analyses, and the differences were considered statistically significant when nominal *p* < 0.05.

### Ethics Approval

All animal experiments were conducted in accordance with the relevant guidelines and regulations approved by the Animal Care Committee of Sunnybrook Research Institute. The approval number for animal experiments is AUP#22‐224.

## Conflict of Interest

The authors declare no conflict of interest.

## Supporting information

Supporting Information

Supporting Information

Supporting Information

## Data Availability

The data that support the findings of this study are available from the corresponding author upon reasonable request.

## References

[1] J. I. Lehtimaki , E. K. Rajakyla , S. Tojkander , P. Lappalainen , Elife 2021, 10. 10.7554/eLife.60710 PMC7877910 33506761

[2] C. Xie , Y. Jiang , Z. Zhu , S. Huang , W. Li , G. Ou , Proc. Natl. Acad. Sci. U. S. A. 2021, 118. 10.1073/pnas.2100805118 PMC8449360 34507987

[3] N. Wu , J. Xu , W. W. Du , X. Li , F. M. Awan , F. Li , S. Misir , E. Eshaghi , J. Lyu , L. Zhou , K. Zeng , A. Adil , S. Wang , B. B. Yang , Mol. Ther. 2021, 29, 1138. 33279723 10.1016/j.ymthe.2020.12.004 PMC7934790

[4] L. Bozal‐Basterra , M. Gonzalez‐Santamarta , V. Muratore , N. Martin‐Martin , A. Ercilla , J. A. Rodriguez , A. Carracedo , J. D. Sutherland , R. Barrio , Front. Cell Dev. Biol. 2021, 9, 624089. 33869174 10.3389/fcell.2021.624089 PMC8049182

[5] S. Duarte , A. Viedma‐Poyatos , E. Navarro‐Carrasco , A. E. Martinez , M. A. Pajares , D. Perez‐Sala , Nat. Commun. 2019, 10, 4200. 31519880 10.1038/s41467-019-12029-4 PMC6744490

[6] S. J. Del Signore , C. F. Kelley , E. M. Messelaar , T. Lemos , M. F. Marchan , B. Ermanoska , M. Mund , T. G. Fai , M. Kaksonen , A. A. Rodal , Elife 2021, 10. 10.7554/eLife.69597 PMC8321554 34324418

[7] S. Tomii , T. Akashi , N. Ando , T. Tamura , A. Sakurai , A. Terada , A. Furukawa , Y. Suzuki , K. Kayamori , K. Sakamoto , et al., Pathobiology 2017, 84, 171. 28002815 10.1159/000452838

[8] A. Chen , P. D. Arora , C. A. McCulloch , A. Wilde , Nat. Commun. 2017, 8, 1530. 29146911 10.1038/s41467-017-01231-x PMC5691081

[9] M. A. Titus , Cold Spring Harb. Perspect. Biol. 2018, 10. 10.1101/cshperspect.a021972 PMC5830894 29496823

[10] A. I. Bachir , A. R. Horwitz , W. J. Nelson , J. M. Bianchini , Cold Spring Harb. Perspect. Biol. 2017, 9. 10.1101/cshperspect.a023234 PMC5495059 28679638

[11] D. M. Mwangangi , E. Manser , R. C. Robinson , Sci. Adv. 2021, 7. 10.1126/sciadv.abd5271 PMC7840138 33571120

[12] B. Zheng , M. Han , M. Bernier , J. K. Wen , FEBS J. 2009, 276, 2669. 19459931 10.1111/j.1742-4658.2009.06986.x PMC2978034

[13] V. Hurst , K. Shimada , S. M. Gasser , Trends Cell Biol. 2019, 29, 462. 30954333 10.1016/j.tcb.2019.02.010

[14] B. J. Belin , T. Lee , R. D. Mullins , eLife 2015, 4, e07735. 26287480 10.7554/eLife.07735 PMC4577826

[15] C. P. Caridi , C. D'Agostino , T. Ryu , G. Zapotoczny , L. Delabaere , X. Li , V. Y. Khodaverdian , N. Amaral , E. Lin , A. R. Rau , et al., Nature 2018, 559, 54. 29925946 10.1038/s41586-018-0242-8 PMC6051730

[16] B. R. Schrank , T. Aparicio , Y. Li , W. Chang , B. T. Chait , G. G. Gundersen , M. E. Gottesman , J. Gautier , Nature 2018, 559, 61. 29925947 10.1038/s41586-018-0237-5 PMC6145447

[17] M. Plessner , R. Grosse , Curr. Opin. Cell Biol. 2019, 56, 1. 30193156 10.1016/j.ceb.2018.08.005

[18] T. Viita , S. Kyheroinen , B. Prajapati , J. Virtanen , M. J. Frilander , M. Varjosalo , M. K. Vartiainen , J. Cell Sci. 2019, 132. 10.1242/jcs.226852 PMC6503952 30890647

[19] M. Sokolova , H. M. Moore , B. Prajapati , J. Dopie , L. Merilainen , M. Honkanen , R. C. Matos , M. Poukkula , V. Hietakangas , M. K. Vartiainen , iScience 2018, 9, 63. 30384134 10.1016/j.isci.2018.10.010 PMC6214840

[20] H. Q. Le , S. Ghatak , C. Y. Yeung , F. Tellkamp , C. Gunschmann , C. Dieterich , A. Yeroslaviz , B. Habermann , A. Pombo , C. M. Niessen , et al., Nat. Cell Biol. 2016, 18, 864. 27398909 10.1038/ncb3387

[21] L. Figard , L. Zheng , N. Biel , Z. Xue , H. Seede , S. Coleman , I. Golding , A. M. Sokac , Cell Rep. 2019, 26, 3493. 30917306 10.1016/j.celrep.2019.02.092 PMC6447309

[22] J. Liu , Q. Xiao , J. Xiao , C. Niu , Y. Li , X. Zhang , Z. Zhou , G. Shu , G. Yin , Signal Transduct. Target Ther. 2022, 7, 3. 34980884 10.1038/s41392-021-00762-6 PMC8724284

[23] C. D. Buckley , J. Tan , K. L. Anderson , D. Hanein , N. Volkmann , W. I. Weis , W. J. Nelson , A. R. Dunn , Science 2014, 346, 1254211. 25359979 10.1126/science.1254211 PMC4364042

[24] T. Valenta , G. Hausmann , K. Basler , EMBO J. 2012, 31, 2714. 22617422 10.1038/emboj.2012.150 PMC3380220

[25] X. P. Xu , S. Pokutta , M. Torres , M. F. Swift , D. Hanein , N. Volkmann , W. I. Weis , Elife 2020, 9. 10.7554/eLife.60878 PMC7588230 32915141

[26] F. Drees , S. Pokutta , S. Yamada , W. J. Nelson , W. I. Weis , Cell 2005, 123, 903. 16325583 10.1016/j.cell.2005.09.021 PMC3369825

[27] W. Wang , Q. Wen , J. Luo , S. Chu , L. Chen , L. Xu , H. Zang , M. M. Alnemah , J. Li , J. Zhou , et al., Theranostics 2017, 7, 2134. 28656063 10.7150/thno.17665 PMC5485425

[28] S. Yamazaki , K. Yamamoto , P. de Lanerolle , M. Harata , Histochem. Cell Biol. 2016, 145, 389. 26900020 10.1007/s00418-016-1416-9

[29] E. Sanchez‐Tillo , O. de Barrios , L. Siles , M. Cuatrecasas , A. Castells , A. Postigo , Proc. Natl. Acad. Sci. U. S. A. 2011, 108, 19204. 22080605 10.1073/pnas.1108977108 PMC3228467

[30] A. Hyrskyluoto , M. K. Vartiainen , Curr. Opin. Cell Biol. 2020, 64, 18. 32088545 10.1016/j.ceb.2020.01.012

[31] M. Plessner , M. Melak , P. Chinchilla , C. Baarlink , R. Grosse , J. Biol. Chem. 2015, 290, 11209. 25759381 10.1074/jbc.M114.627166 PMC4416828

[32] A. Sasaki , Y. Masuda , Y. Ohta , K. Ikeda , K. Watanabe , J. Biol. Chem. 2001, 276, 17871. 11278410 10.1074/jbc.M008422200

[33] M. Lederer , B. M. Jockusch , M. Rothkegel , J. Cell Sci. 2005, 118, 331. 15615774 10.1242/jcs.01618

[34] E. J. Schmidt , S. Funes , J. E. McKeon , B. R. Morgan , S. Boopathy , L. C. O'Connor , O. Bilsel , F. Massi , A. Jegou , D. A. Bosco , Proc. Natl. Acad. Sci. U. S. A. 2021, 118. 10.1073/pnas.2024605118 PMC8201830 34074767

[35] B. Felipe‐Abrio , A. Carnero , Cancers 2020, 12. 10.3390/cancers12010254 PMC7017249 31968688

[36] B. Felipe‐Abrio , E. M. Verdugo‐Sivianes , C. Saez , A. Carnero , Cancers 2019, 11. 10.3390/cancers11020235 PMC6406377 30781655

[37] X. Xu , Y. Xia , J. Ma , W. Li , N. Niu , X. Li , H. Tao , J. Xu , X. He , Int. J. Oncol. 2020, 57, 522. 32468020 10.3892/ijo.2020.5072 PMC7307585

[38] E. Wustenhagen , F. Boukhallouk , I. Negwer , K. Rajalingam , F. Stubenrauch , L. Florin , Oncogene 2018, 37, 6275. 30018400 10.1038/s41388-018-0398-6 PMC6265261

[39] M. Chatzifrangkeskou , D. E. Pefani , M. Eyres , I. Vendrell , R. Fischer , D. Pankova , E. O'Neill , EMBO J. 2019, 38, 101168. 10.15252/embj.2018101168 PMC6694222 31414556

[40] C. Odaka , M. L. Sanders , P. Crews , Clin. Diagn. Lab. Immunol. 2000, 7, 947. 11063504 10.1128/cdli.7.6.947-952.2000 PMC95991

[41] I. Maluykova , O. Gutsal , M. Laiko , A. Kane , M. Donowitz , O. Kovbasnjuk , Biochim. Biophys. Acta 2008, 1782, 370. 18342638 10.1016/j.bbadis.2008.01.010 PMC2509583

[42] S. R. White , P. Williams , K. R. Wojcik , S. Sun , P. S. Hiemstra , K. F. Rabe , D. R. Dorscheid , Am. J. Respir. Cell Mol. Biol. 2001, 24, 282. 11245627 10.1165/ajrcmb.24.3.3995

[43] C. Baarlink , H. Wang , R. Grosse , Science 2013, 340, 864. 23558171 10.1126/science.1235038

[44] R. C. Stone , I. Pastar , N. Ojeh , V. Chen , S. Liu , K. I. Garzon , M. Tomic‐Canic , Cell Tissue Res. 2016, 365, 495. 27461257 10.1007/s00441-016-2464-0 PMC5011038

[45] D. Haensel , X. Dai , Dev. Dyn. 2018, 247, 473. 28795450 10.1002/dvdy.24561 PMC5809211

[46] L. Liu , X. Liu , X. Ren , Y. Tian , Z. Chen , X. Xu , Y. Du , C. Jiang , Y. Fang , Z. Liu , B. Fan , Q. Zhang , G. Jin , X. Yang , X. Zhang , Sci. Rep. 2016, 6, 21602. 26905010 10.1038/srep21602 PMC4764856

[47] D. V. Iwamoto , A. Huehn , B. Simon , C. Huet‐Calderwood , M. Baldassarre , C. V. Sindelar , D. A. Calderwood , Nat. Struct. Mol. Biol. 2018, 25, 918. 30224736 10.1038/s41594-018-0128-3 PMC6173970

[48] L. Attisano , S. T. Lee‐Hoeflich , Genome Biol. 2001, 2, reviews3010.1. 11532220 10.1186/gb-2001-2-8-reviews3010 PMC138956

[49] B. Felipe‐Abrio , E. M. Verdugo‐Sivianes , A. Carnero , Mol. Oncol. 2019, 13, 1519. 31066170 10.1002/1878-0261.12499 PMC6599841

[50] M. Coccia , A. Rossi , A. Riccio , E. Trotta , M. G. Santoro , Proc. Natl. Acad. Sci. U. S. A. 2017, 114, 1045. 28096332 10.1073/pnas.1616112114 PMC5293105

[51] M. Nourbakhsh , H. Hauser , EMBO J. 1999, 18, 6415. 10562553 10.1093/emboj/18.22.6415 PMC1171704

[52] Z. Lu , Y. Li , A. Takwi , B. Li , J. Zhang , D. J. Conklin , K. H. Young , R. Martin , Y. Li , EMBO J. 2011, 30, 57. 21113131 10.1038/emboj.2010.296 PMC3020116

[53] J. Pernier , S. Shekhar , A. Jegou , B. Guichard , M. F. Carlier , Dev. Cell 2016, 36, 201. 26812019 10.1016/j.devcel.2015.12.024 PMC4729542

[54] Z. G. Yang , F. M. Awan , W. W. Du , Y. Zeng , J. Lyu , D. Wu , S. Gupta , W. Yang , B. B. Yang , Mol Ther 2017, 25, 2062. 28676341 10.1016/j.ymthe.2017.05.022 PMC5589065

