# Supplementary material for: Nuclear Actin Polymerization Regulates Cell Epithelial‐Mesenchymal Transition
Source: Adv Sci (Weinh). 2023 Aug 11;10(28):2300425. doi: 10.1002/advs.202300425 (PMC10558697; doi:10.1002/advs.202300425)
Supplement: Supplementary file 1 — Supporting Information [file ADVS-10-2300425-s003.pdf]

ADVANCED SCIENCE
Open Access

Supporting Information

for *Adv. Sci.*, DOI 10.1002/advs.202300425

Nuclear Actin Polymerization Regulates Cell Epithelial-Mesenchymal Transition

*William W. Du, Javeria Qadir, Kevin Y. Du, Yu Chen, Nan Wu and Burton B. Yang**

Supporting Information

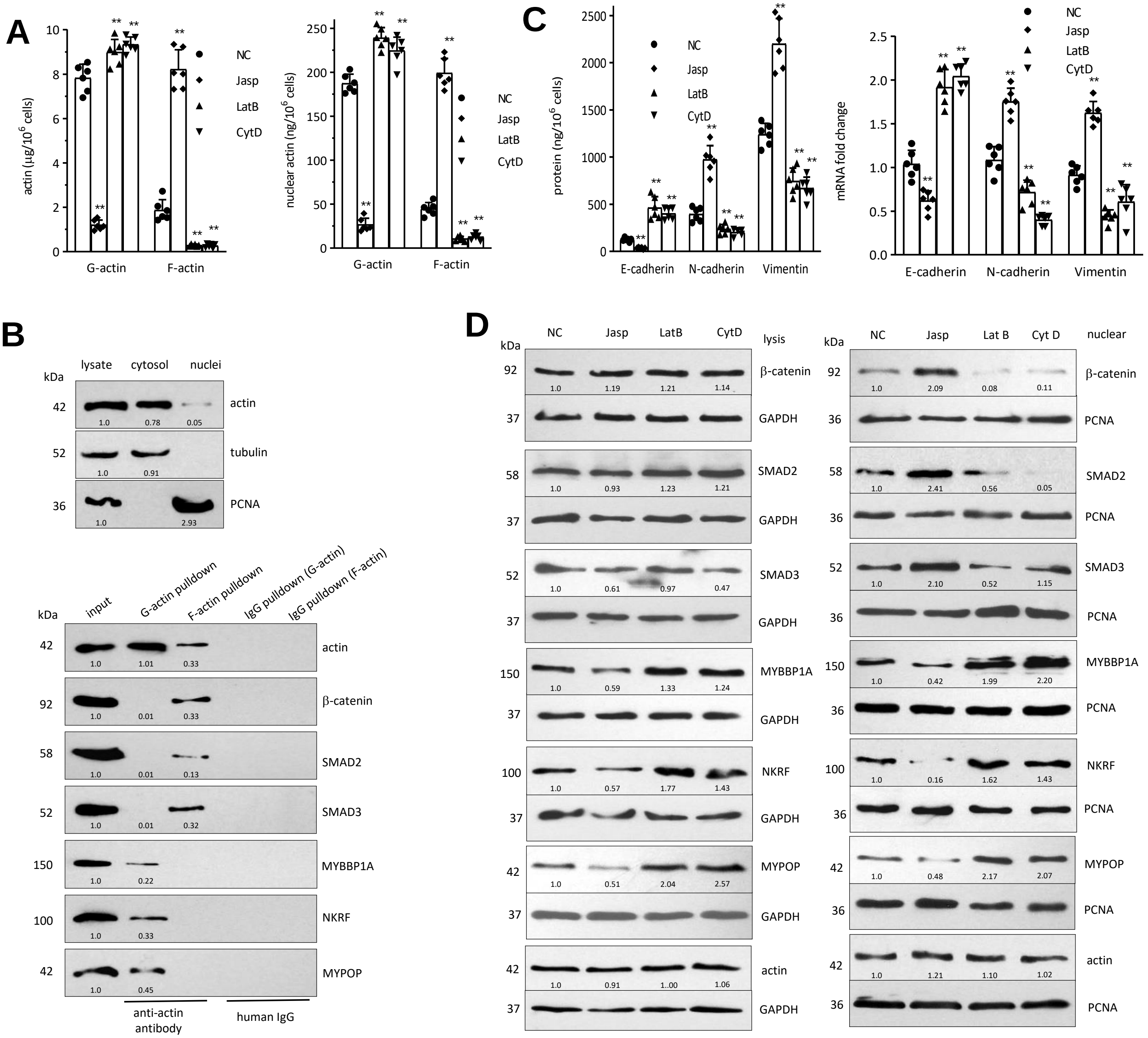

Fig S1. Jasp, LatB and CytD regulate nuclear actin dynamics and EMT.

A, HEK 293T cells were cultured in 0.5 μM Jasp, 1 μM LatB or 10 μM CytD for 1 h and subjected to nuclear F/G-actin fractionation and ELISA. Jasp induced actin polymerization, while LatB and CytD induced actin depolymerization in total cells (left) and nuclei (right).

B, Upper, Cell lysate, cytoplasmic, and nuclear fraction were subjected to Western blot, probed with antibodies against actin, tubulin (marker for cytosol), and PCNA (marker for nuclei). Lower, Cell lysate (input) was subjected to isolation of nuclear G-actin and F-actin, both of which were precipitated with anti-actin antibody and human IgG, respectively. MYBBP1A, NKRF and MYPOP were co-precipitated with G-actin, while β-catenin, SMAD2 and SMAD3 were co-precipitated with F-actin.

C, Left, ELISA analysis showed that Jasp treatment repressed E-cadherin, enhanced N-cadherin and vimentin. Whereas, LatB and CytD treatment increased E-cadherin, decreased N-cadherin and vimentin protein levels. ***p<0.01* versus NC (*n=6*). Right, Jasp, LatB and CytD treated HEK 293T cells were subjected to RT-PCR, showing that Jasp treatment repressed E-cadherin, enhanced N-cadherin and vimentin mRNA levels. Whereas, LatB and CytD treatment enhanced E-cadherin, repressed N-cadherin and vimentin mRNA expression levels. ***p<0.01* versus NC (*n=6*).

D, Left, Western blot showed that these chemicals did not change total actin, β-catenin, SMAD2, and SMAD3 expression in the cells. However, Jasp treatment repressed MYBBP1A, NKRF and MYPOP, and LatB and CytD treatment enhanced MYBBP1A, NKRF and MYPOP expression. Right, The nuclear extracts were lysed and subjected to Western blot, showing that the above-mentioned chemicals did not change total actin expression in the nuclei. Jasp treatment enhanced β-catenin, SMAD2, SMAD3, and repressed MYBBP1A, NKRF and MYPOP in the nuclei. LatB and CytD repressed β-catenin, SMAD2, SMAD3, and enhanced MYBBP1A, NKRF and MYPOP expression in the nuclei.

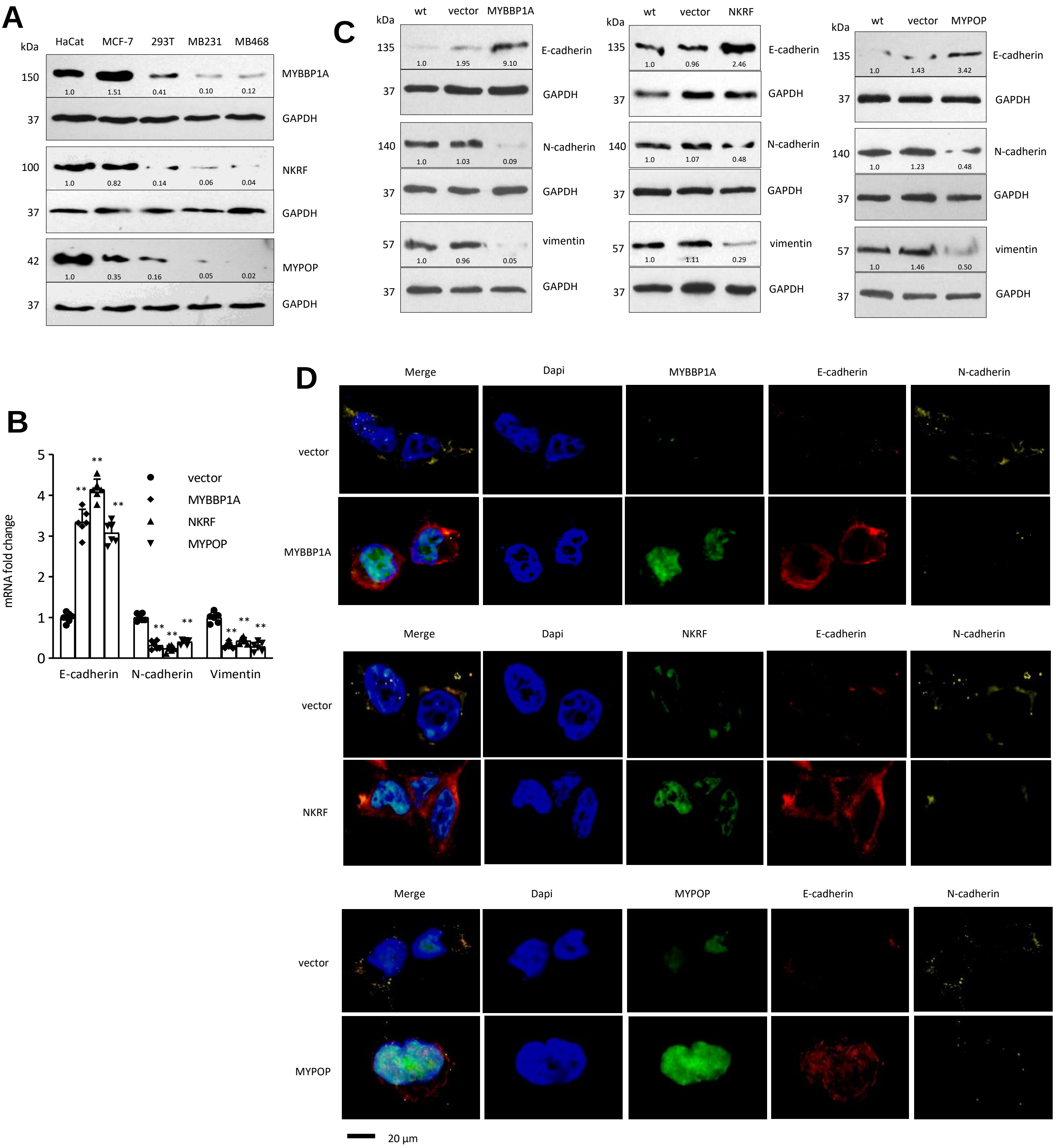

Fig S2. MYBBP1A, NKRF and MYPOP repress EMT associated proteins.

A, Expression of MYBBP1A, NKRF and MYPOP was analyzed on Western blot. While HaCat and MCF-7 cells expressed high levels of these protein, they were expressed at low levels in MDA-MB231 and MDA-MB468 cells, and at moderate levels in HEK-293T cells.

B, HEK 293T cells were transfected with MYBBP1A, NKRF and MYPOP. RT-PCR showed that overexpression of MYBBP1A, NKRF and MYPOP increased E-cadherin, and repressed N-cadherin and vimentin mRNA levels. ***$p<0.01$* versus vector (*n=6*).

C, Western blot showed that overexpression of MYBBP1A, NKRF and MYPOP increased E-cadherin, while it repressed N-cadherin and vimentin expression levels.

D, Immunofluorescence staining showed that MYBBP1A, NKRF or MYPOP (Green) were mainly expressed in the nucleus, and expression of MYBBP1A, NKRF or MYPOP enhanced E-cadherin (red), and repressed N-cadherin (yellow) levels.

A

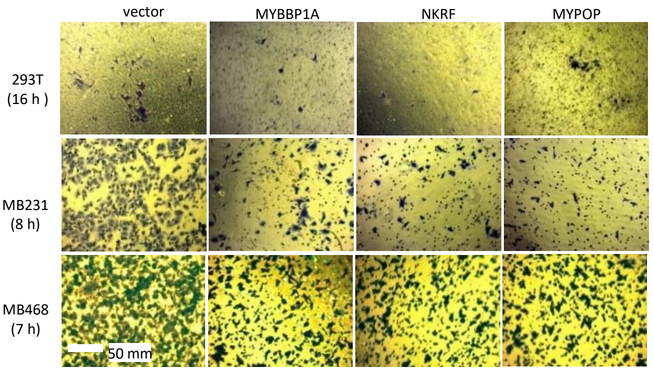

B

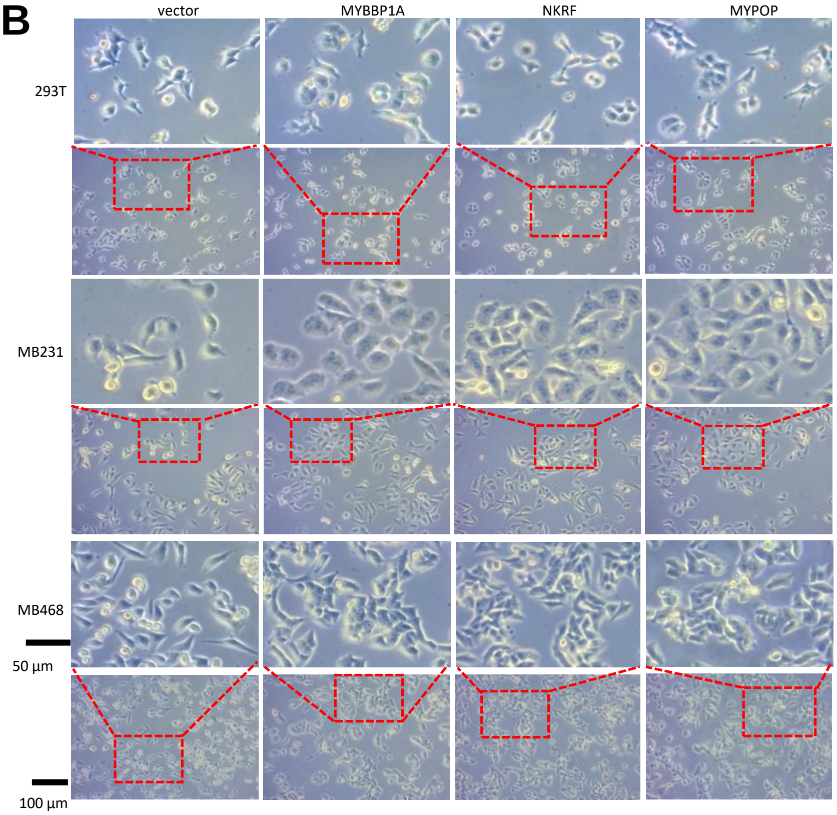

C

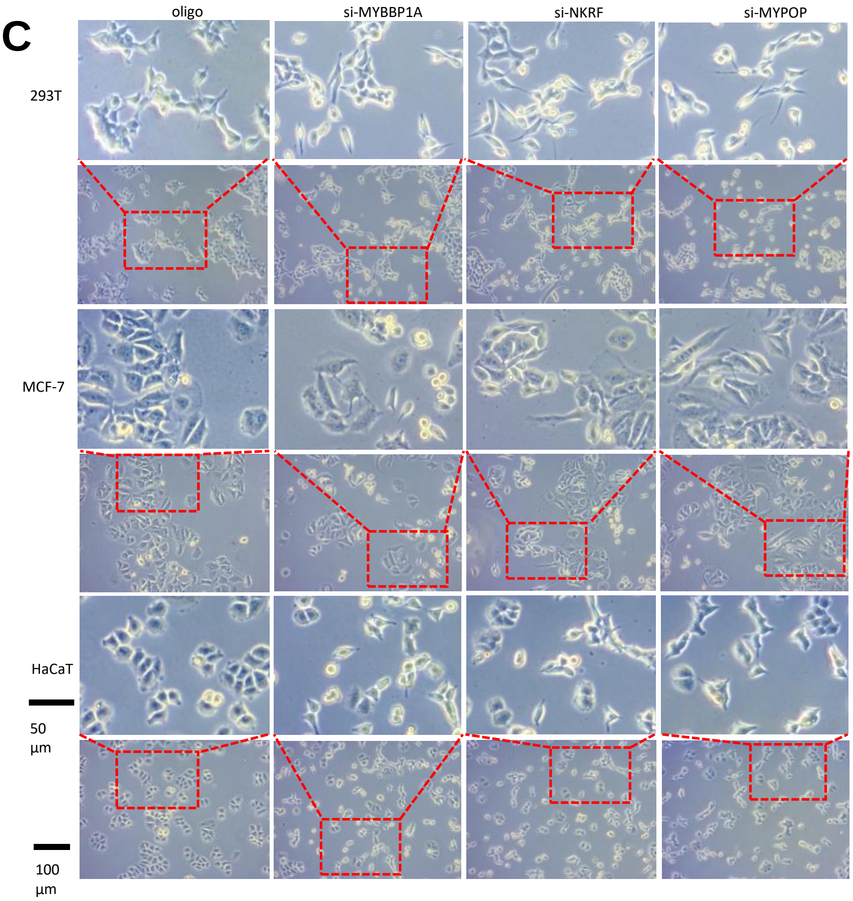

Fig S3. The effects of MYBBP1A, NKRF and MYPOP on cuboidal epithelial migration and morphology.

A, HEK 293T, MDA-MB-231 and MDA-MB468 cells were transfected with MYBBP1A, NKRF or MYPOP, and processed to chamber migration assays for indicated time points. Typical images showed that overexpression of MYBBP1A, NKRF or MYPOP suppressed cell migration.

B, Cultured in basal medium for 24 h, the transfected cells lost elongated mesenchymal shape, and acquired cuboidal epithelial structure.

C, HEK 293T, HaCaT and MCF-7 cells were transfected with MYBBP1A, NKRF or MYPOP siRNAs, and cultured in basal medium for 24 h. MYBBP1A, NKRF or MYPOP siRNA transfected cells lost cuboidal epithelial morphology and displayed elongated mesenchymal shape.

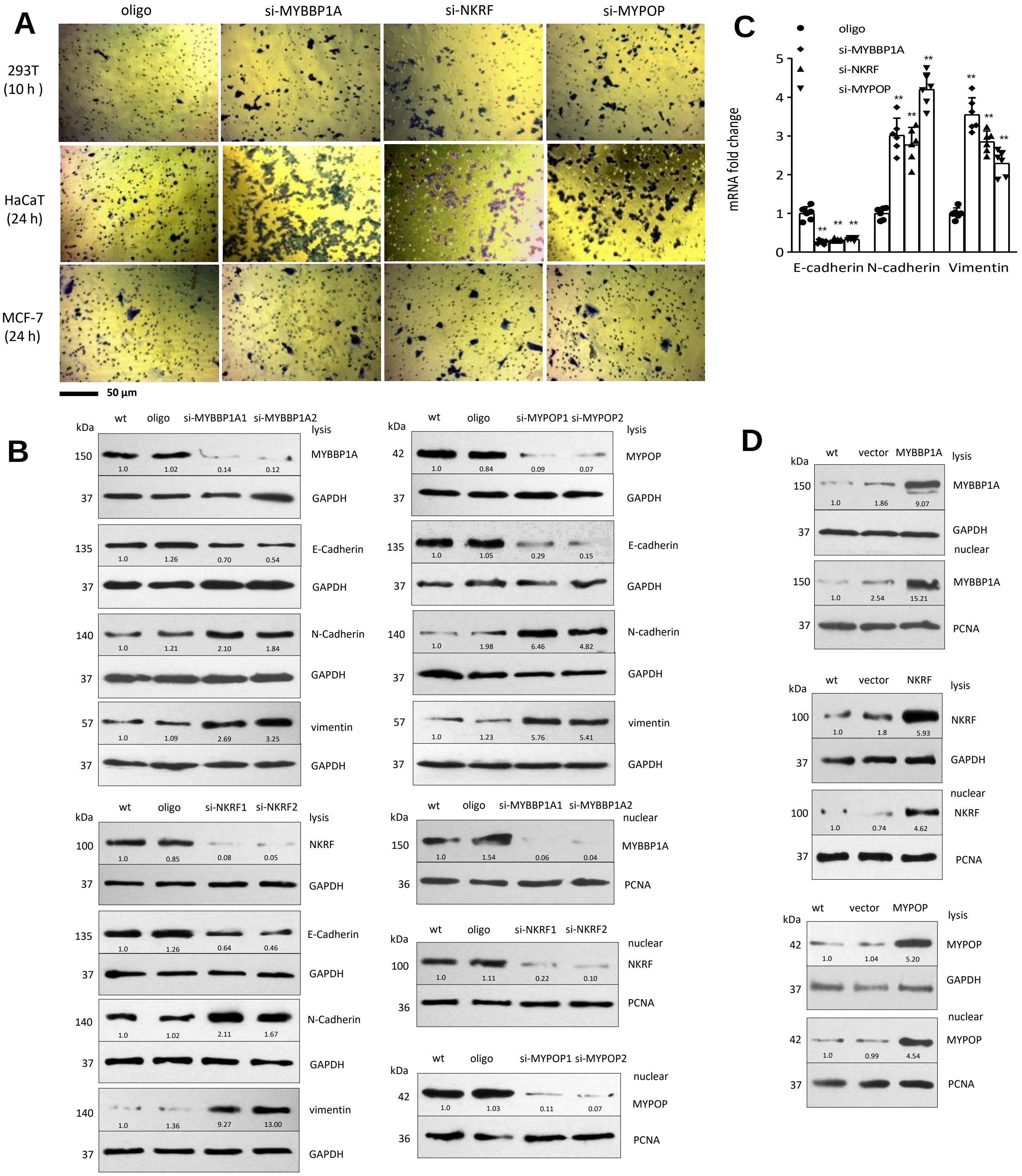

Fig S4. Silencing MYBBP1A, NKRF or MYPOP promotes EMT.

A, HEK 293T, HaCaT and MCF-7 cells were transfected with MYBBP1A, NKRF or MYPOP siRNAs, and processed to chamber migration assays for indicated time points. Typical images showed that silencing MYBBP1A, NKRF or MYPOP enhanced cell migration.

B, Western blot showed that silencing MYBBP1A, NKRF and MYPOP decreased E-cadherin, and increased N-cadherin and vimentin expression.

C, RT-PCR showed that silencing MYBBP1A, NKRF or MYPOP decreased E-cadherin, and increased N-cadherin and vimentin mRNA levels. **$p<0.01$ versus oligo ($n=6$).

D, The transfected cells were subjected to nuclear fractionation. Western blot showed that overexpression of the aforementioned transcription factors increased their expression levels in both cells and nuclei.

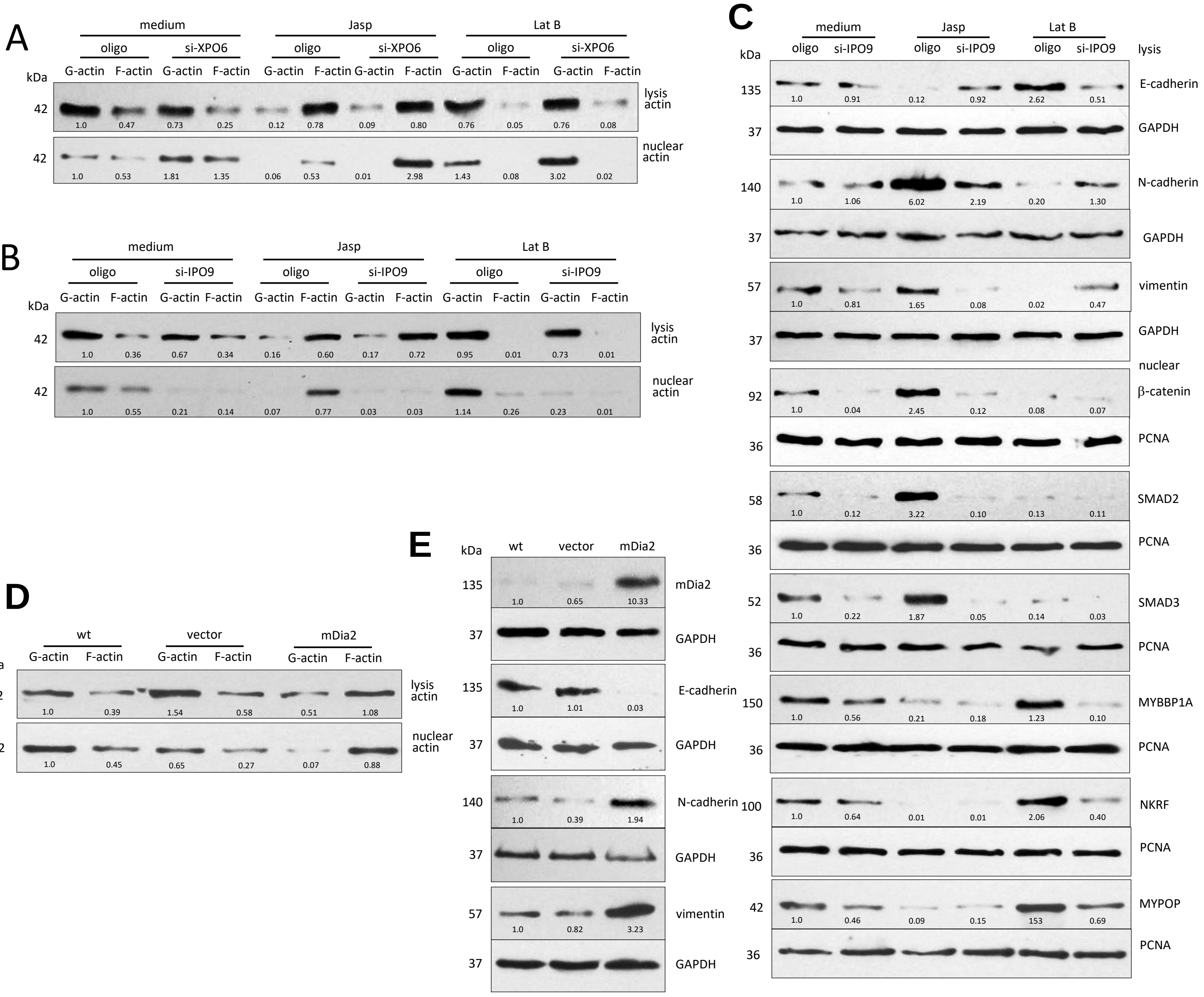

Fig S5. Increase of nuclear F-actin promotes EMT.

A, HEK293T cells were transfected with XPO6 siRNAs or the control oligo, and cultured in 0.1 μM Jasp or 0.1 μM LatB for 24 h. The cells and nuclear extracts were lysed with LAS2 and subjected to F-actin/G-actin fractionation. Western blot showed that medium/si-XPO6 increased both F-actin and G-actin in the nuclei compared to the medium/oligo cells. Jasp/si-XPO6 cells showed same cell actin dynamics and nuclear G-actin levels as Jasp/oligo, and increased F-actin in the nuclei. LatB/si-XPO6 cells showed same cell actin dynamics and nuclear F-actin levels as LatB/oligo, and increased G-actin in the nuclei.

B, HEK293T cells were transfected with IPO9 siRNAs or the control oligo and cultured in 0.1 μM Jasp or 0.1 μM LatB for 24 h. Western blot showed that the medium/si-IPO9 cells displayed decrease in both F-actin and G-actin in the nuclei compared to the medium/oligo cells. Jasp/si-IPO9 cells showed same cell actin dynamics and nuclear G-actin levels as Jasp/oligo, but decreased F-actin in the nuclei. LatB/si-IPO9 cells showed same cell actin dynamics and nuclear F-actin levels as LatB/oligo, but decreased G-actin in the nuclei.

C, Upper, HEK293T cells were transfected with IPO9 siRNAs or the control oligo, cultured in 0.1 μM Jasp or 0.1 μM LatB for 24 h and subjected to Western blot. Jasp/IPO9- cells showed increased E-cadherin, and repressed N-cadherin and vimentin compared to Jasp/oligo, while LatB/IPO9- cells displayed decreased E-cadherin, and increased N-cadherin and vimentin expression compared to LatB/oligo. Lower, The nuclear extracts from the above-mentioned cells were subjected to Western blot. Jasp/IPO9- cells showed repressed β-catenin, SMAD2 and SMAD3 expression in the nuclei compared to Jasp/oligo, while LatB/IPO9- cells displayed decreased MYBBP1A, NKRF and MYPOP expression in the nuclei compared to LatB/oligo.

D, HEK 293T cells were transfected with the control vector or mDia2 and subjected to F-actin/G-actin fractionation. Western blot showed that expression of mDia2 enhanced actin polymerization in the cells and the nuclei.

E, These cells were then subjected to Western blot, showing that overexpression of mDia2 repressed E-cadherin, and enhanced N-cadherin and vimentin.

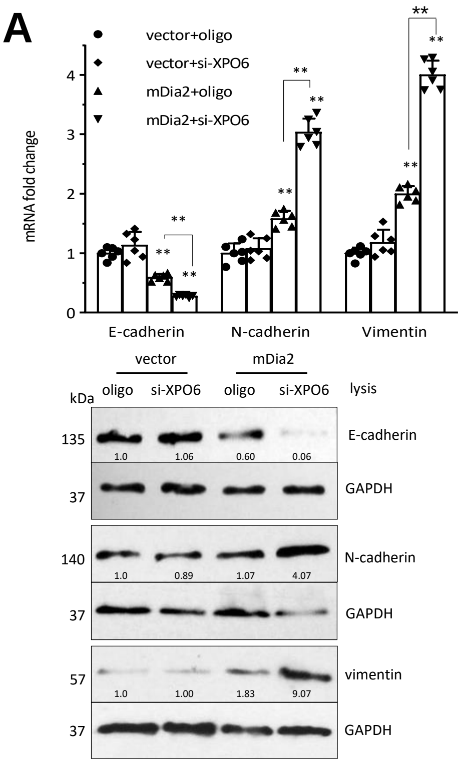

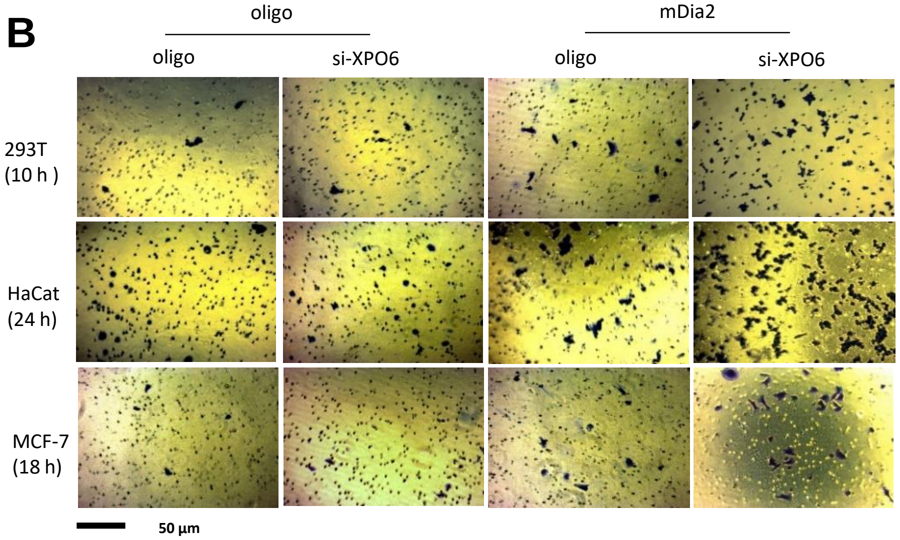

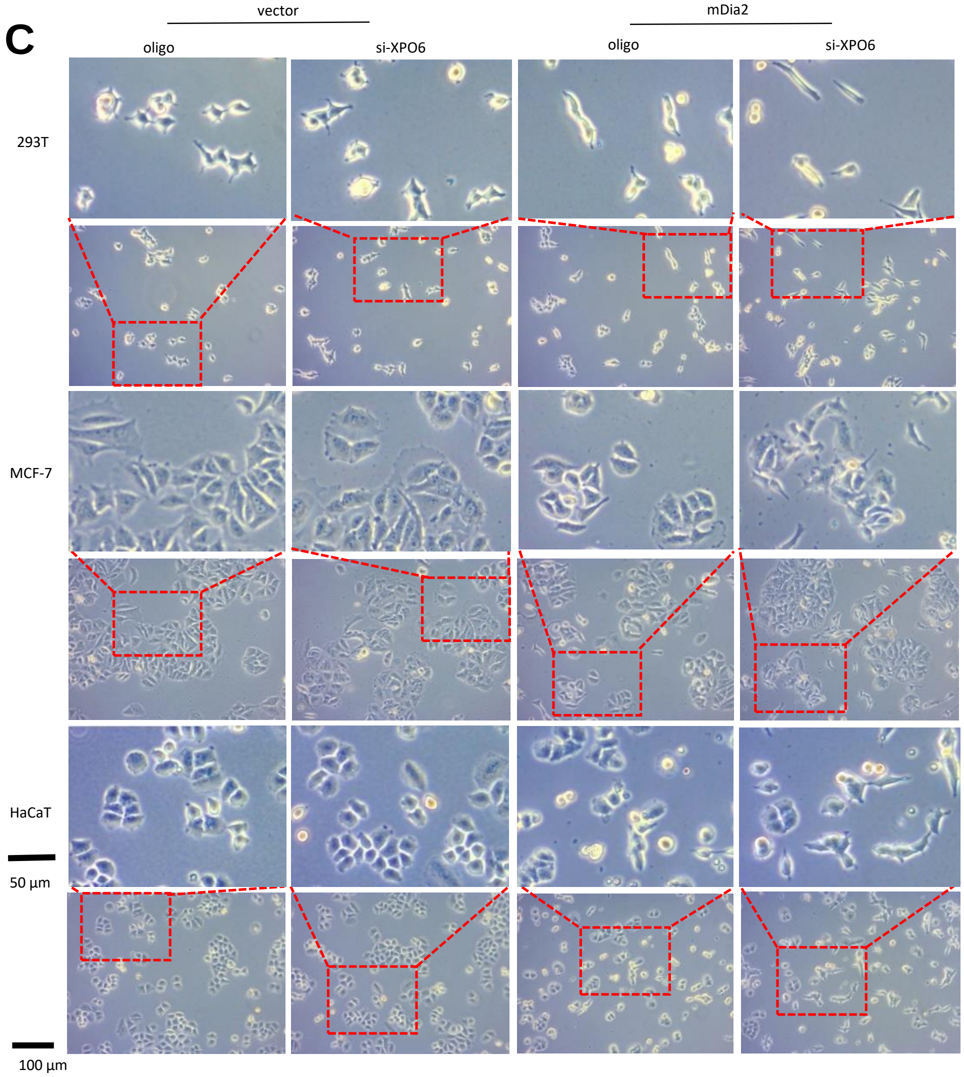

Fig S6. Increase of nuclear F-actin induces elongated mesenchymal cell morphology.

A, HEK293T cells were transfected with mDia2 with or without XPO6 siRNAs and subjected to RT-PCR. mDia2+/XPO6- cells showed decreased E-cadherin, and increased N-cadherin and vimentin mRNA (upper) and protein (lower) levels compared to mDia2+/oligo. ***p<0.01* versus oligo (*n=6*).

B, HEK 293T, HaCaT and MCF-7 cells were co-transfected with mDia2 and XPO6 siRNAs and processed to chamber migration assays for indicated time points. Typical images showed that mDia2+/XPO6- cells displayed enhanced cell migration compared to mDia2+/oligo treated cells.

C, HEK 293T, HaCaT and MCF-7 cells were co-transfected with mDia2 and XPO6 siRNAs, and cultured in basal medium for 24 h, showing that mDia2+/XPO6- cells displayed elongated mesenchymal morphology.

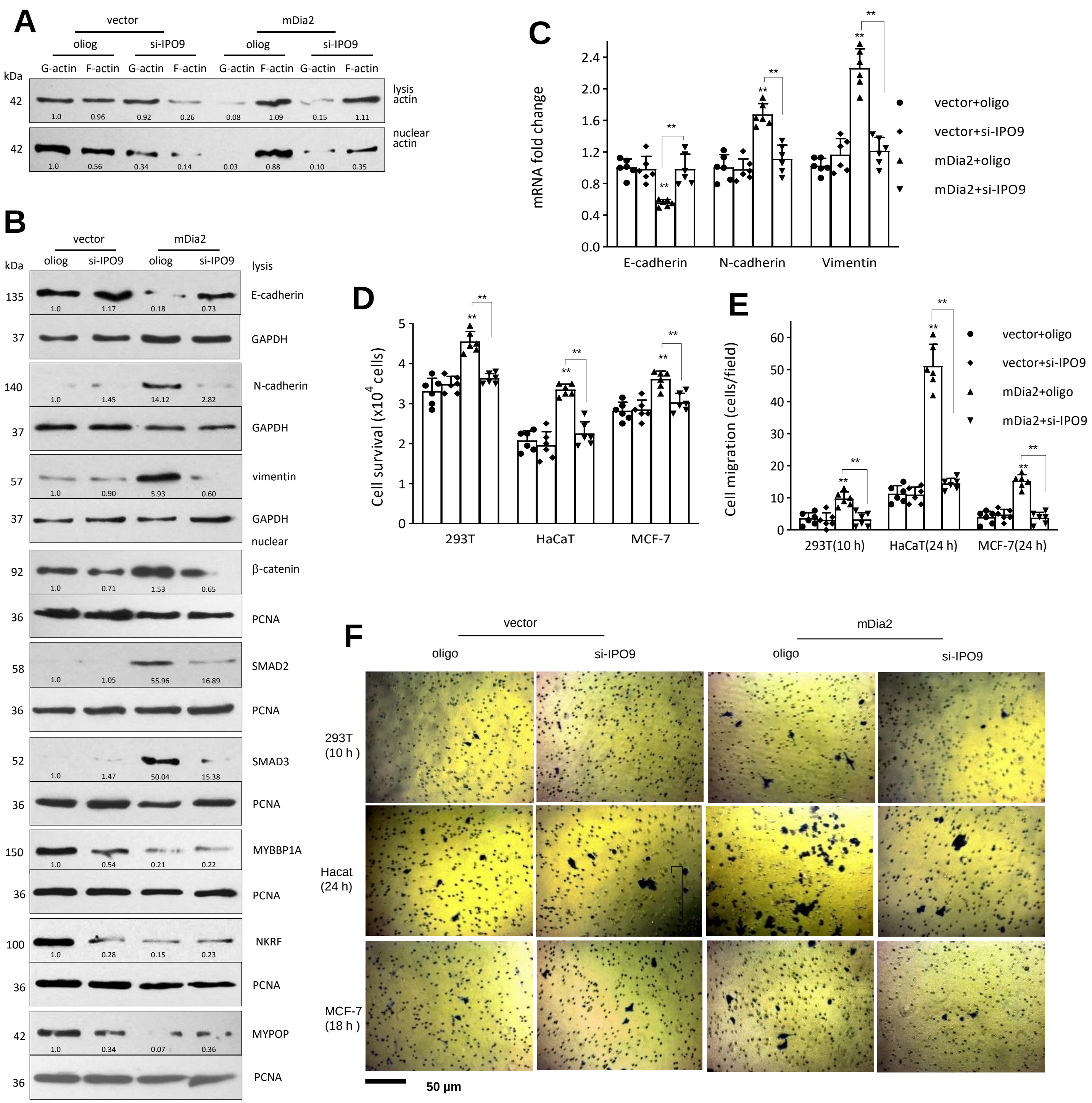

Fig S7. Decrease of nuclear F-actin represses EMT.

A, HEK 293T cells were transfected with the control vector or mDia2 with or without IPO9 siRNAs. Western blot showed that expression of mDia2 enhanced actin polymerization in the cells and the nuclei, and silencing IPO9 did not change F-actin/G-actin in the cells. Silencing IPO9 repressed nuclear F-actin levels in mDia2 transfected cells.

B, mDia2+/IPO9- cells showed enhanced E-cadherin, and repressed N-cadherin and vimentin expression compared to mDia2+/oligo. mDia2+/IPO9- cells showed repressed β-catenin, SMAD2 and SMAD3 expression in the nuclei compared to mDia2+/oligo.

C, RT-PCR showed that mDia2+/IPO9- cells expressed increased E-cadherin, and decreased N-cadherin and vimentin compared to mDia2+/oligo cells. ***$p<0.01$* versus oligo (*n=6*).

D, HEK 293T, HaCaT and MCF-7 cells were co-transfected with mDia2 and IPO9 siRNAs and cultured in basal medium with 650 μM H_2O_2 for 24 h, showing that mDia2+/IPO9- cells displayed decreased cell survival. ***$p<0.01$* versus oligo (*n=6*).

E, Graphs showed that mDia2+/IPO9- cells displayed repressed cell migration compared to mDia2+/oligo cells. ***$p<0.01$* versus oligo (*n=6*).

F, HEK 293T, HaCaT and MCF-7 cells were co-transfected with mDia2 and IPO9 siRNAs and processed to chamber migration assays for indicated time points. Typical images showing cell migration assays are presented.

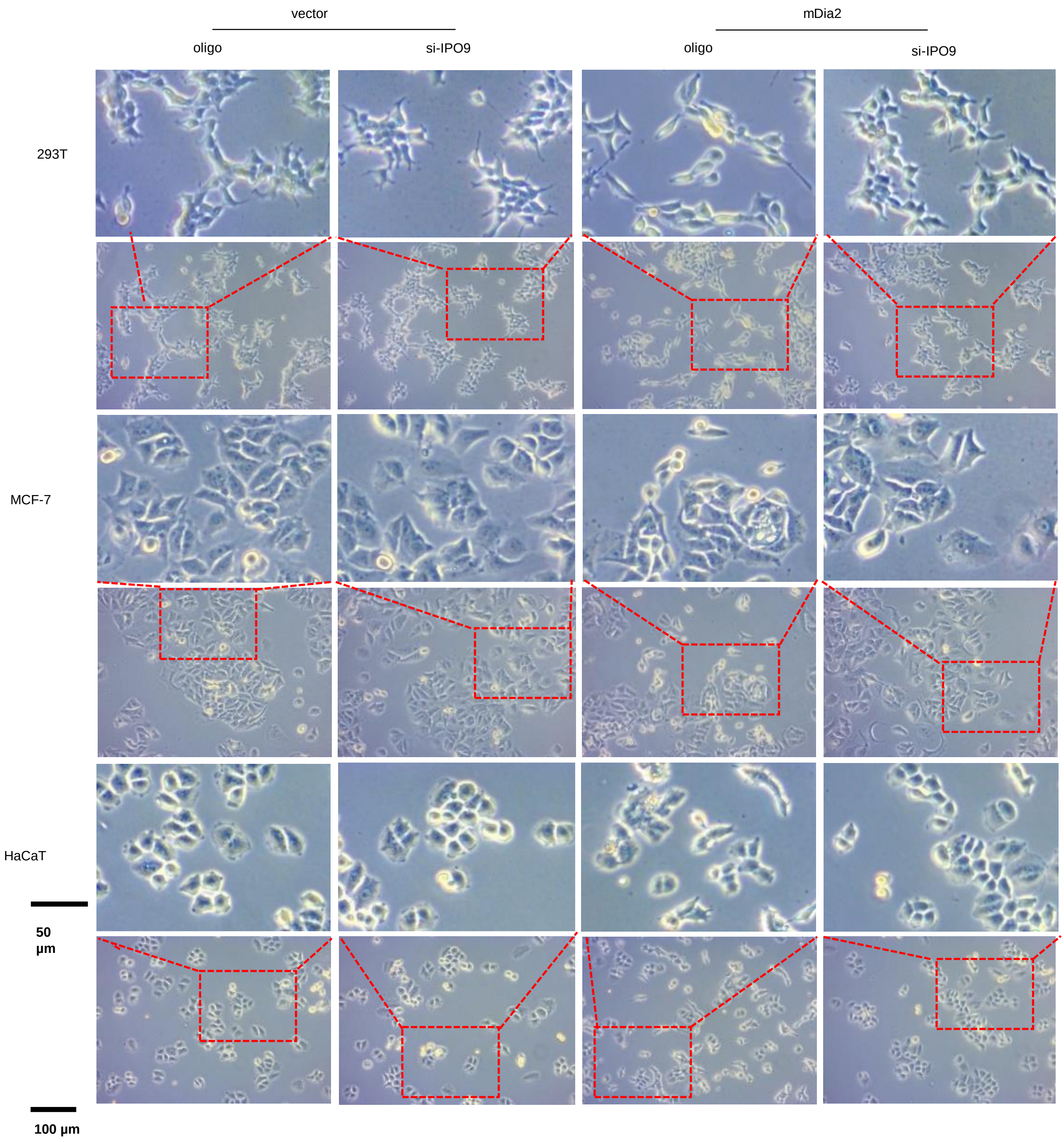

Fig S8. Decrease of nuclear F-actin induces cuboidal epithelial cell shape.
HEK 293T, HaCaT and MCF-7 cells were co-transfected with mDia2 and IPO9 siRNAs, and cultured in basal medium for 24 h, showing that mDia2+/IPO9- cells displayed cuboidal epithelial shape compared to mDia2+/oligo cells.

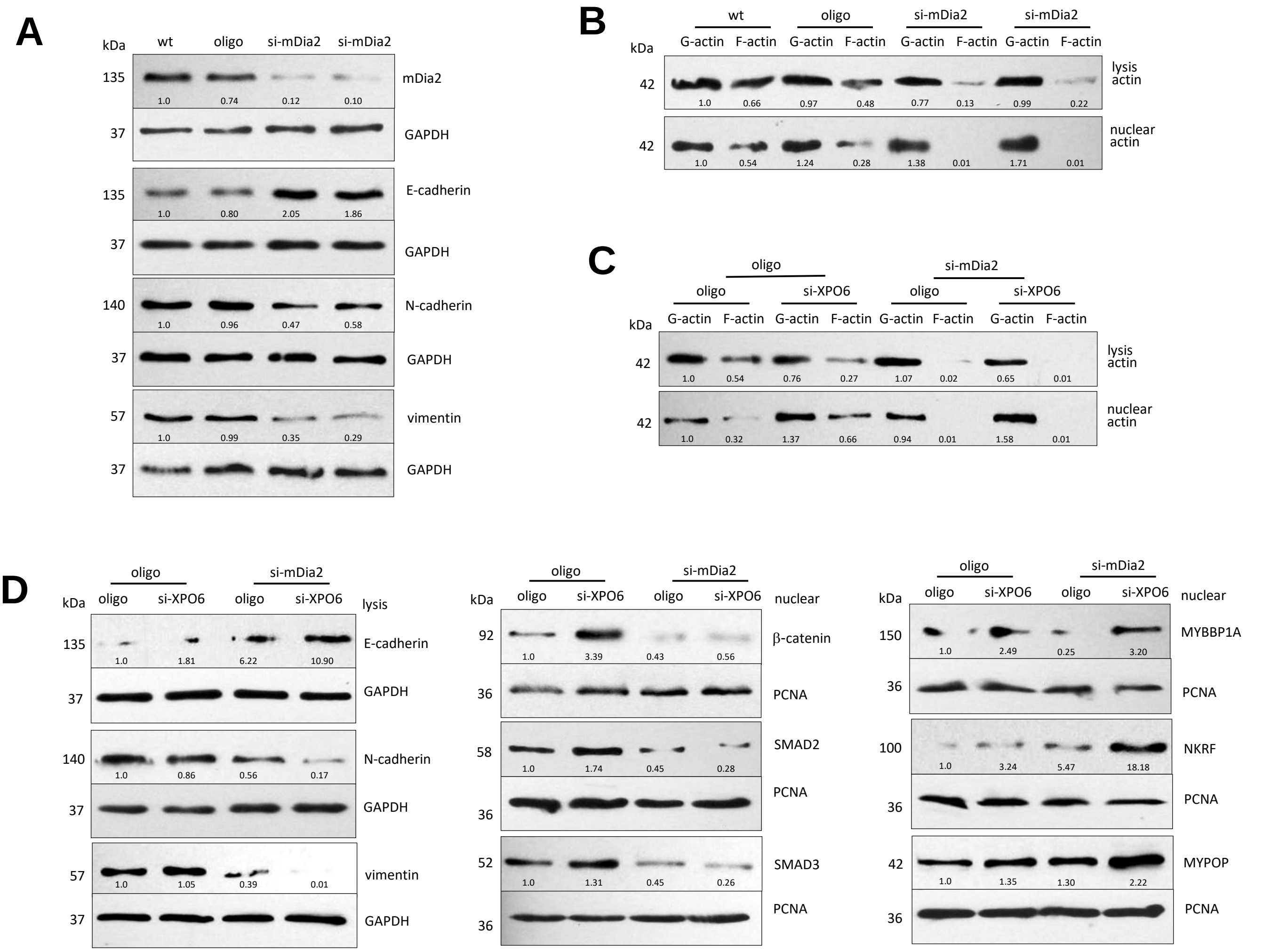

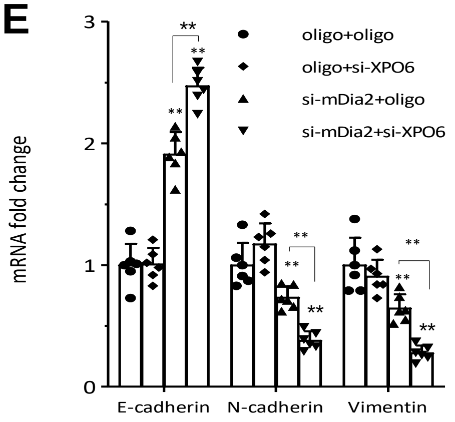

Fig S9. Increase of nuclear G-actin represses EMT.

A, Silencing mDia2 repressed actin polymerization. HEK 293T cells were transfected with the control oligo or mDia2 siRNAs. Western blot showed that silencing mDia2 with siRNAs decreased cell mDia2, N-cadherin and vimentin, and increased E-cadherin expression.

B, The cell lysates and nuclear extracts were subjected to F-actin/G-actin fractionation. Western blot showed that silencing mDia2 repressed cell and nuclear actin polymerization.

C, HEK 293T cells were transfected with the control oligo or mDia2 siRNAs with or without XPO6 siRNAs, and subjected to F-actin/G-actin fractionation. Western blot showed that mDia2-/XPO6- cells expressed increased nuclear G-actin, and same cell actin dynamics and nuclear F-actin as mDia2-/oligo cells.

D, Left, Western blot showed that mDia2-/XPO6- cells expressed increased E-cadherin, and decreased N-cadherin and vimentin compared to mDia2-/oligo. Middle and right, mDia2-/XPO6- cells showed increased MYBBP1A, NKRF and MYPOP levels in the nuclei compared to mDia2-/oligo.

E, Aforementioned transfected cells were subjected to RT-PCR. mDia2-/XPO6- cells showed increased E-cadherin, and decreased N-cadherin and vimentin mRNA levels compared to mDia2-/oligo cells.

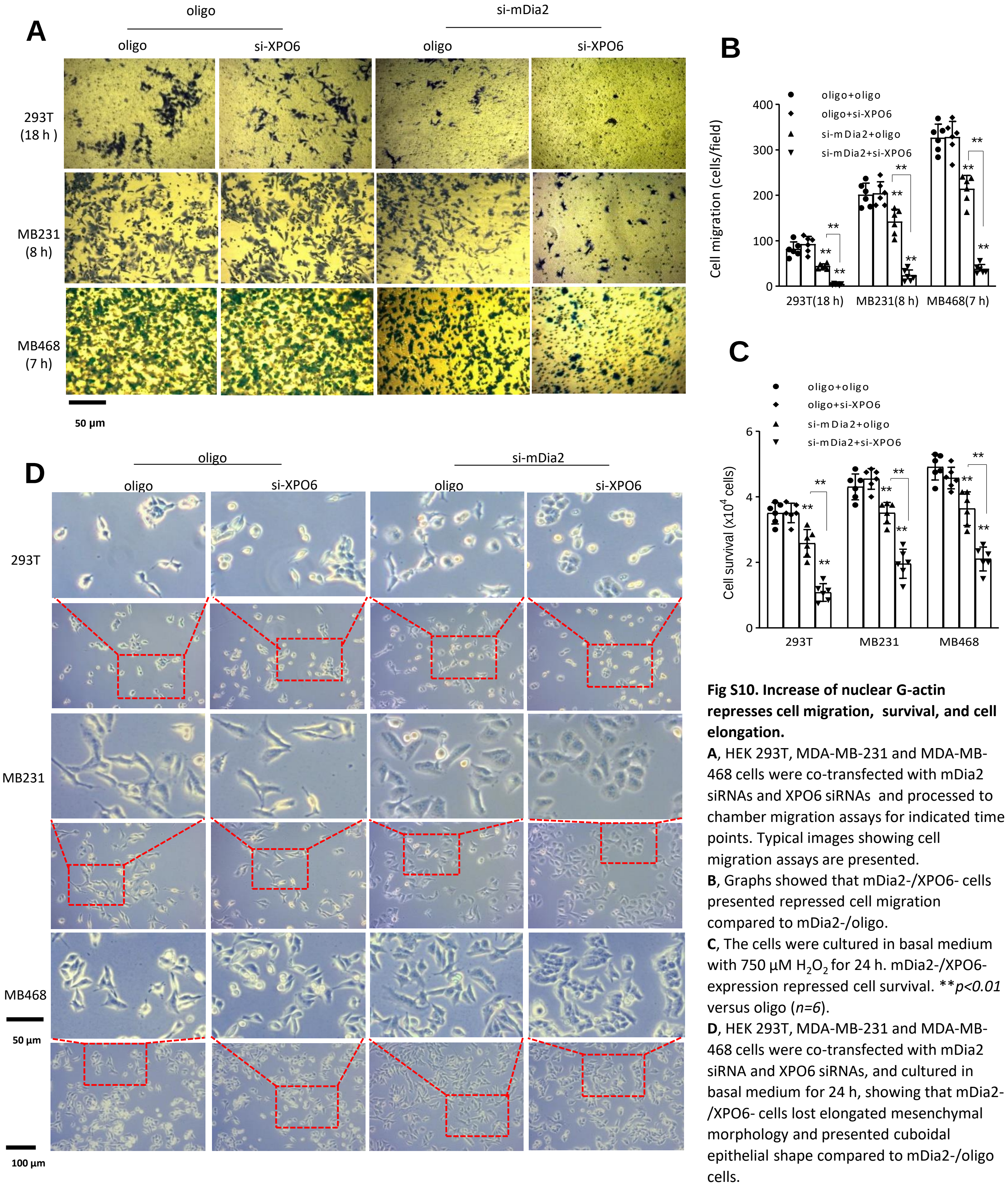

Fig S10. Increase of nuclear G-actin represses cell migration, survival, and cell elongation.

A, HEK 293T, MDA-MB-231 and MDA-MB-468 cells were co-transfected with mDia2 siRNAs and XPO6 siRNAs and processed to chamber migration assays for indicated time points. Typical images showing cell migration assays are presented.

B, Graphs showed that mDia2-/XPO6- cells presented repressed cell migration compared to mDia2-/oligo.

C, The cells were cultured in basal medium with 750 μM H_2O_2 for 24 h. mDia2-/XPO6- expression repressed cell survival. ***p<0.01* versus oligo (*n=6*).

D, HEK 293T, MDA-MB-231 and MDA-MB-468 cells were co-transfected with mDia2 siRNA and XPO6 siRNAs, and cultured in basal medium for 24 h, showing that mDia2-/XPO6- cells lost elongated mesenchymal morphology and presented cuboidal epithelial shape compared to mDia2-/oligo cells.

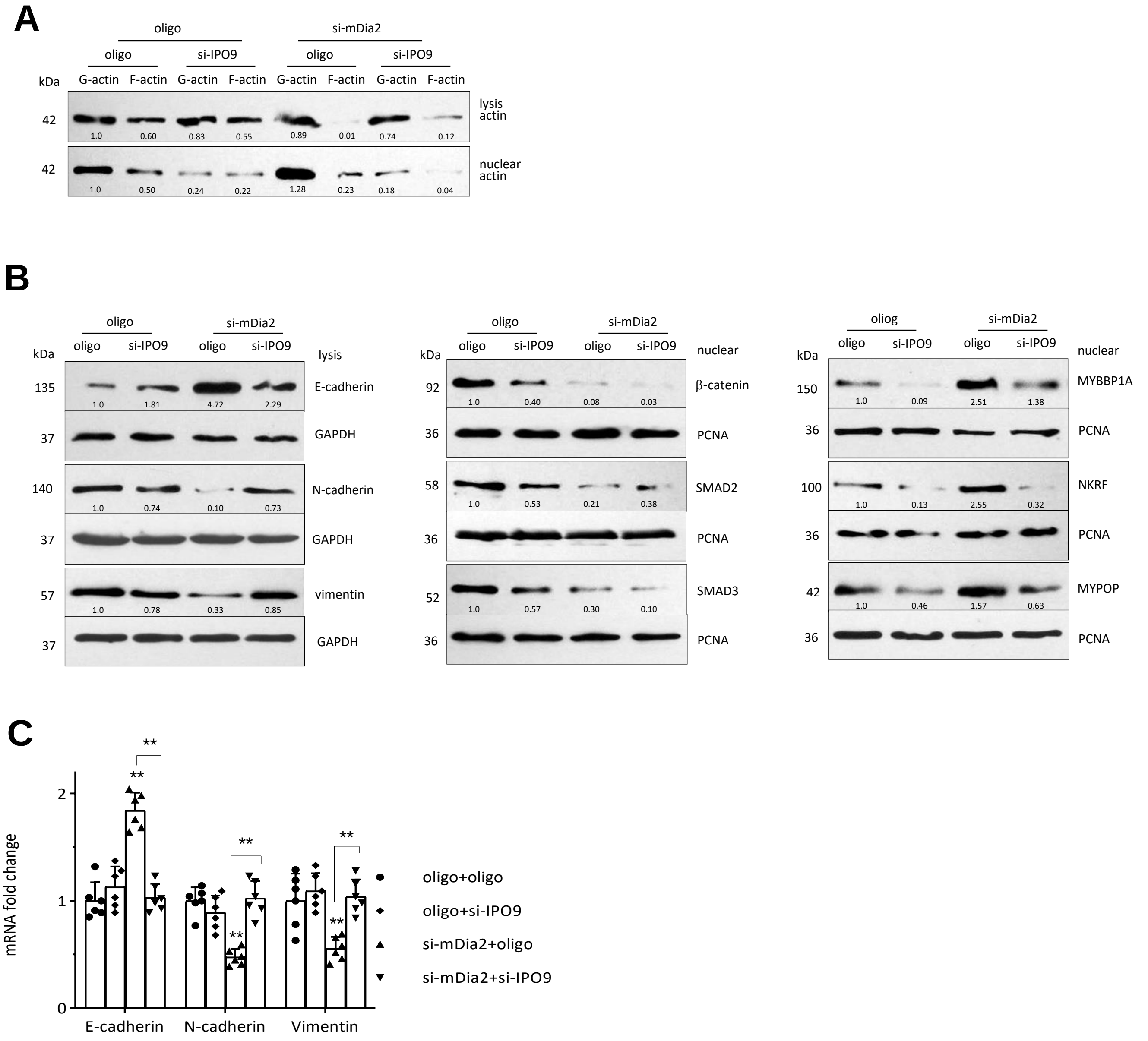

Fig S11. Decrease of nuclear G-actin enhances EMT.

A, HEK 293T cells were transfected with the control oligo or mDia2 siRNAs with or without IPO9 siRNAs. The cell and nuclear extract lysates were subjected to F-actin/G-actin fractionation. Western blot showed that mDia2-/IPO9- cells exhibited same cell actin dynamics and nuclear F-actin expression as mDia2-/oligo cells, but decreased nuclear G-actin levels.

B, The cell lysates and nuclear extracts were subjected to Western blot, showing that mDia2-/IPO9- cells presented decreased E-cadherin, and increased N-cadherin and vimentin expression compared to mDia2-/oligo cells. Moreover, mDia2-/IPO9- cells showed decreased MYBBP1A, NKRF and MYPOP expression in the nuclei.

C, RT-PCR showed that mDia2-/IPO9- cells presented decreased E-cadherin, and increased N-cadherin and vimentin mRNA levels compared to mDia2-/oligo cells. ***$p<0.01$* versus oligo (*$n=6$*).

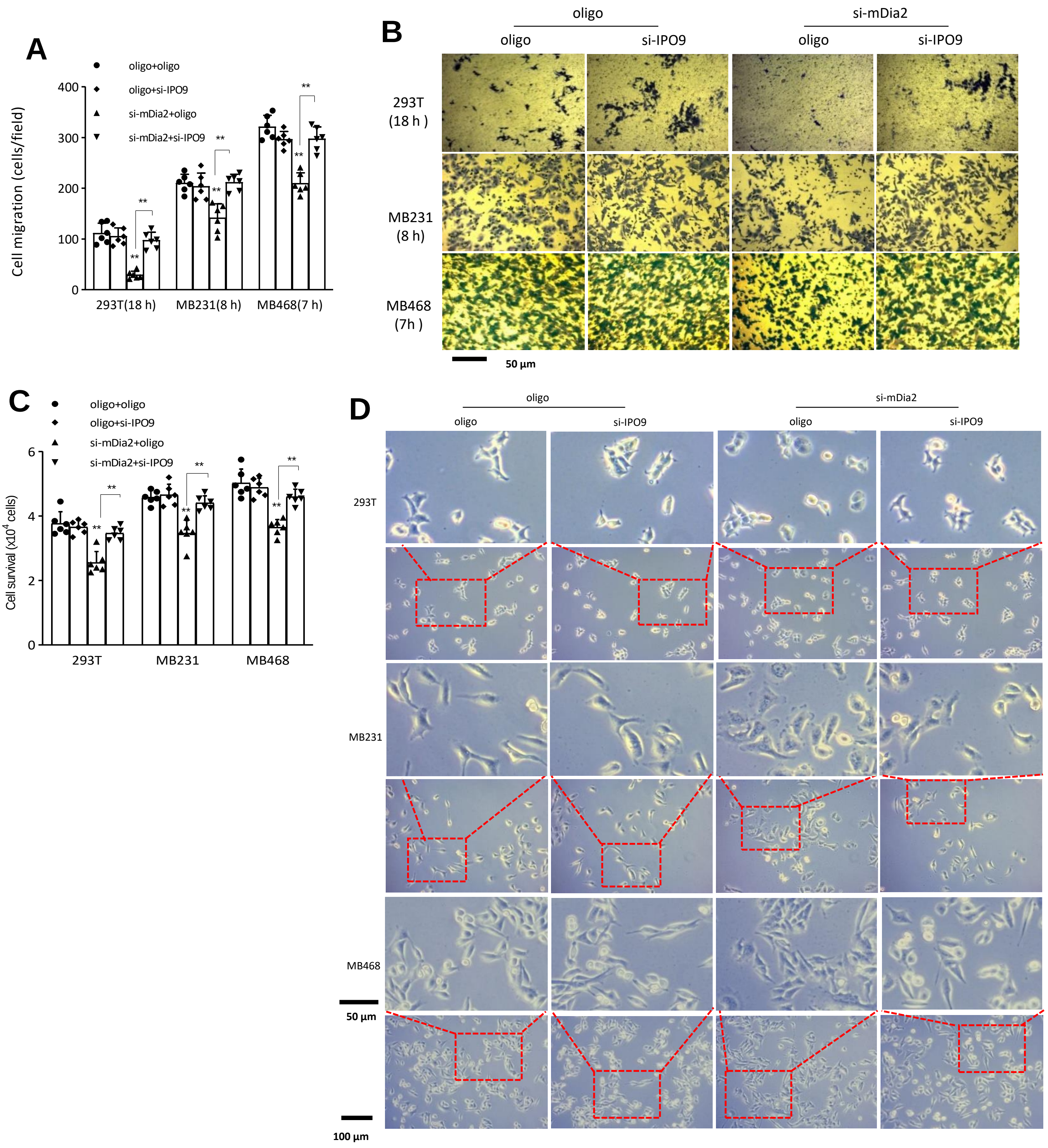

Fig S12. Decrease of nuclear G-actin enhances cell migration, survival, and cell elongation.

A, HEK 293T, MDA-MB-231 and MDA-MB-468 cells were co-transfected with mDia2 siRNAs and IPO9 siRNAs and processed to chamber migration assays for indicated time points, showing that mDia2-/IPO9- cells presented enhanced cell migration compared to mDia2-/oligo cells.

B, Typical images showed cell migration assays.

C, The cells were cultured in basal medium with 750 µM H_2O_2 for 24 h. mDia2-/IPO9 expression enhanced cell survival compared to the mDia2-/oligo cells. ***p<0.01* versus oligo (*n=6*).

D, HEK 293T, MDA-MB-231 and MDA-MB-468 cells were co-transfected with mDia2 siRNA and IPO9 siRNAs, and cultured in basal medium for 24 h, showing that mDia2-/IPO9- cells displayed elongated mesenchymal morphology compared to mDia2-/oligo cells.

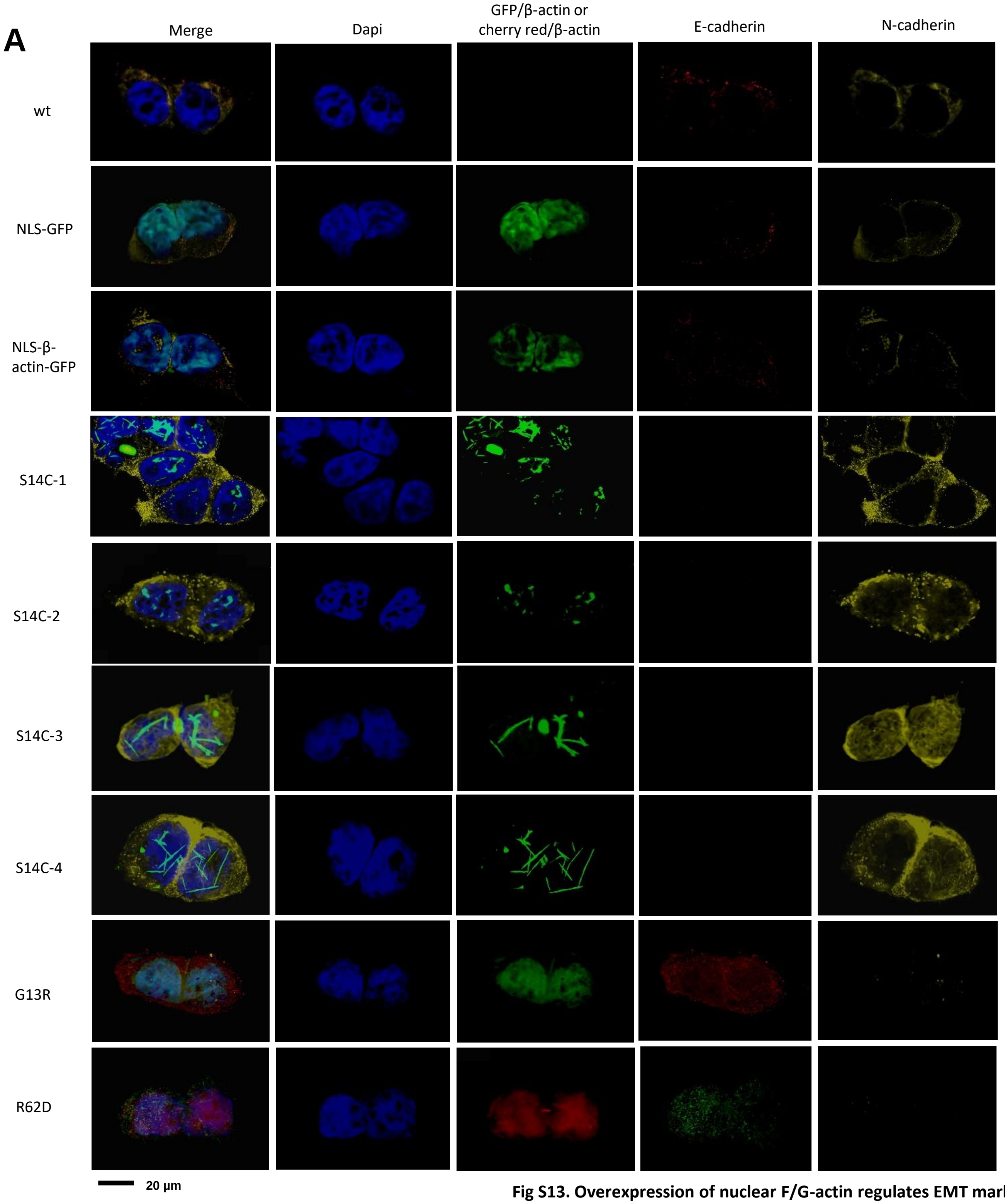

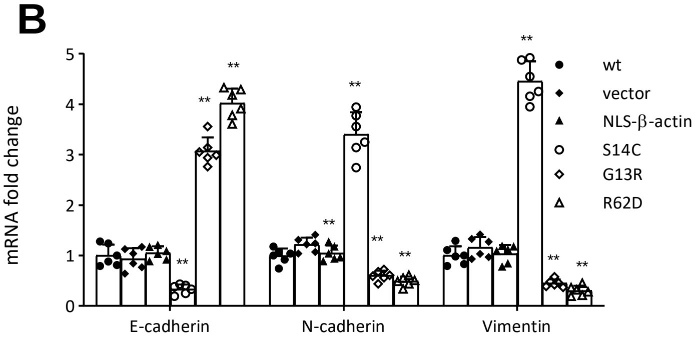

Fig S13. Overexpression of nuclear F/G-actin regulates EMT markers.
A, HEK293T cells were transfected with the control vector, YFP-NLS-β-actin (NLS-β-actin), YFP-NLS-β-actin S14C (S14C), YFP-NLS-β-actin G13R (G13R) or pmCherry-NLS-β-actin R62D (R62D). Immunofluorescence staining showed that transfection with S14C enhanced nuclear F-actin and cell N-cadherin levels, and repressed E-cadherin expression levels. Transfection with G13R or R62D enhanced nuclear G-actin and cell E-cadherin levels, and repressed N-cadherin levels.
B, RT-PCR showed that transfection with S14C repressed E-cadherin, and enhanced N-cadherin and vimentin mRNA levels, while overexpression of G13R or R62D enhanced E-cadherin, and repressed N-cadherin and vimentin mRNA levels. ***p<0.01* versus vector (*n=6*).

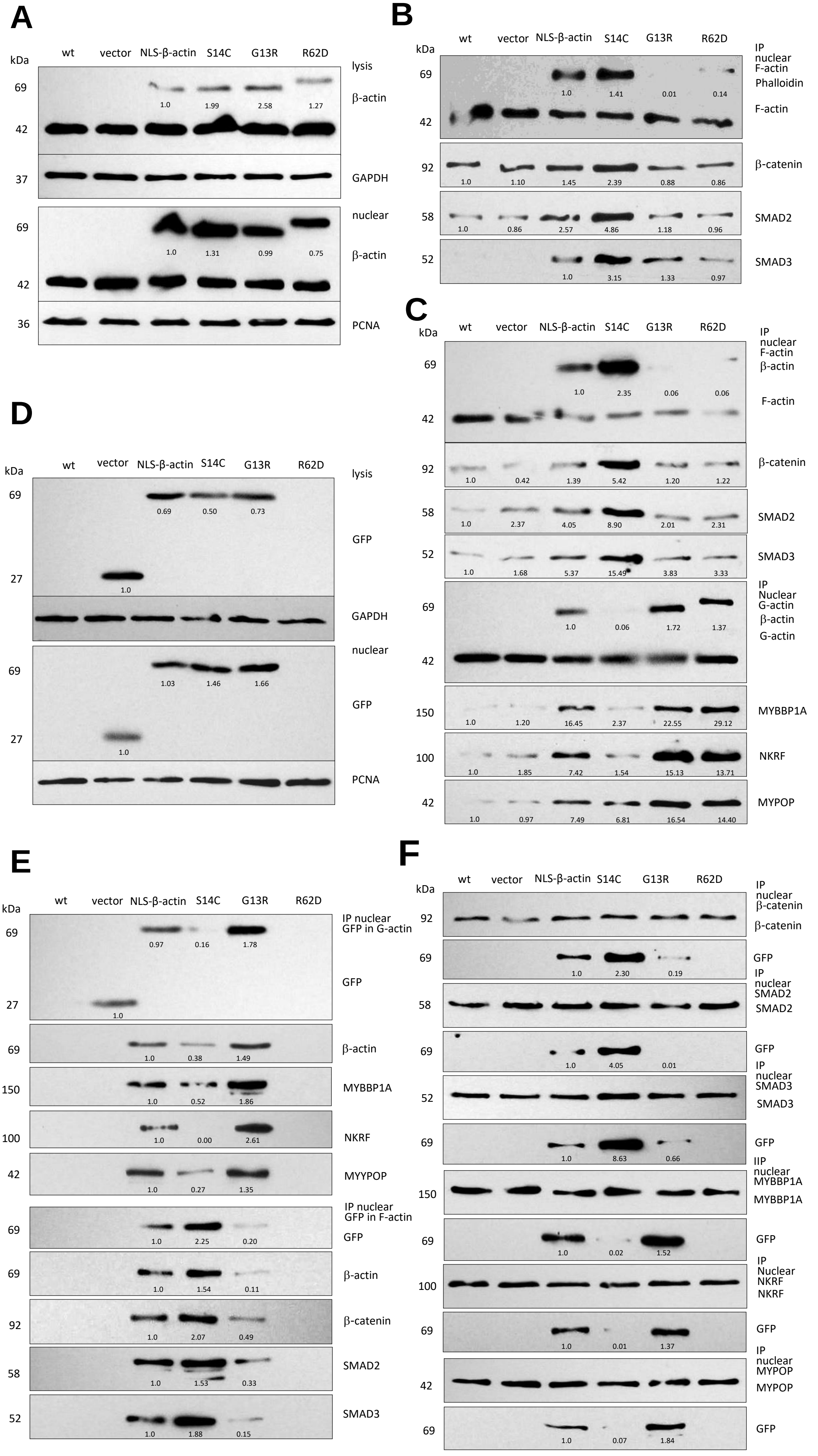

Fig S14. Nuclear F/G-actin interacts with β-catenin, SMAD2, SMAD3, MYBBP1A, NKRF and MYPOP.

A, HEK 293T cells were transfected with the control vector, NLS-β-actin, S14C, G13R or R62D constructs. Western blot showed that transfection with NLS-β-actin, S14C, G13R or R62D constructs expressed β-actin in the cells and the nuclei.

B, The above transfected cell nuclear extracts were subjected to biotin-XX Phalloidin precipitation. Western blot showed that precipitation of nuclear F-actin with Phalloidin pulled down β-catenin, SMAD2 and SMAD3.

C, The above transfected cell nuclear extracts were subjected to F/G-actin fractionation, and immunoprecipitation with antibody against β-actin. Western blot showed that precipitation of G-actin pulled down MYBBP1A, NKRF and MYPOP, while precipitation of F-actin pulled down β-catenin, SMAD2 and SMAD3.

D, The above transfected cells and nuclear extracts were subjected to Western blot, showing the expression of GFP-β-actin fusion protein in the transfected cells.

E, The above transfected cell nuclear extracts were subjected to F/G-actin fractionation, and immunoprecipitation with antibody against GFP. Western blot showed that precipitation of GFP-β-actin (G-actin) pulled down MYBBP1A, NKRF and MYPOP, while precipitation of GFP-β-actin (F-actin) pulled down β-catenin, SMAD2 and SMAD3.

F, The above transfected cell nuclear extracts were subjected to immunoprecipitation with antibodies against β-catenin, SMAD2, SMAD3, MYBBP1A, NKRF and MYPOP. Western blot showed that precipitation of β-catenin, SMAD2 or SMAD3 pulled down GFP-β-actin in NLS-β-actin and S14C, while precipitation of MYBBP1A, NKRF or MYPOP pulled down GFP-β-actin in NLS-β-actin and G13R transfected cells.

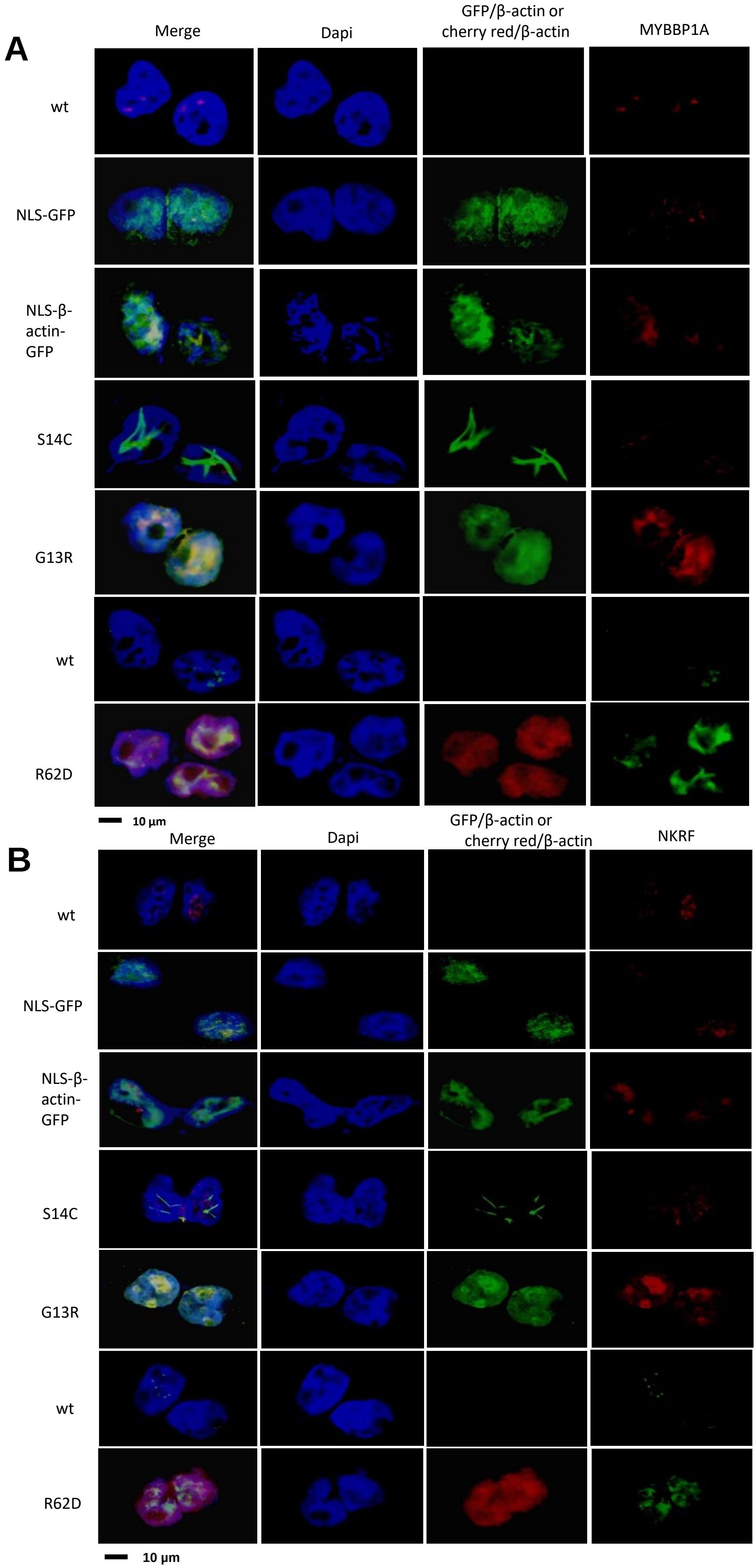

Fig S15. Overexpression of nuclear G-actin enhanced expression of MYBBP1A and NKRF.
A, HEK293T cells were transfected with the control vector, NLS-β-actin, S14C, G13R or R62D. Immunofluorescence staining showed the colocalization of MYBBP1A with the nuclear G-actin in G13R and R62D transfected cells.
B, Immunofluorescence staining showed the colocalization of NKRF with the nuclear G-actin in G13R and R62D transfected cells.

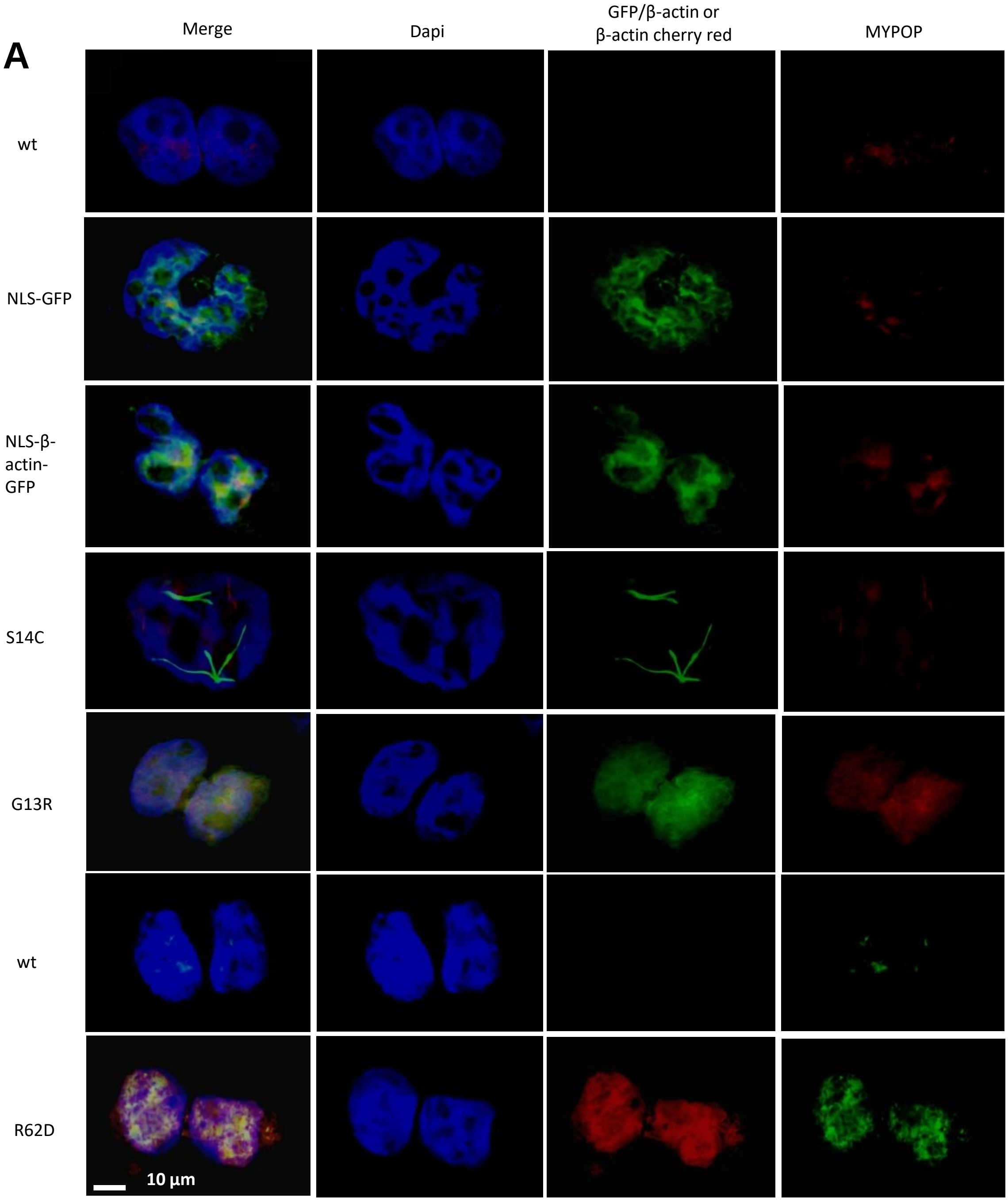

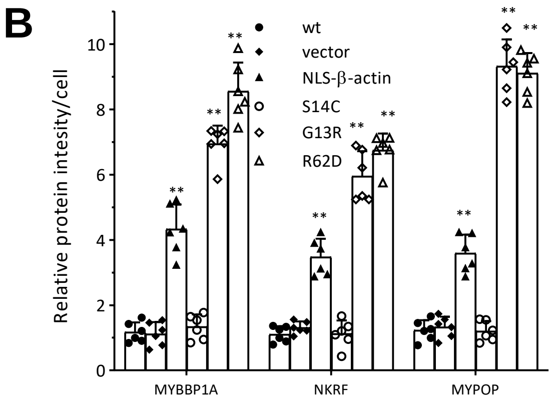

Fig S16. Overexpression of nuclear G-actin enhanced MYPOP expression.

A, HEK293T cells were transfected with the control vector, NLS-β-actin, S14C, G13R or R62D. Immunofluorescence staining showed the colocalization of MYPOP with the nuclear G-actin in G13R and R62D transfected cells.

B, ImageJ analysis showed that transfection with NLS-β-actin, G13R or R62D enhanced cell MYBBP1A, NKRF or MYPOP expression, while transfection with S14C did not affect the expression of these proteins. ***p<0.01* versus vector (*n=6*).

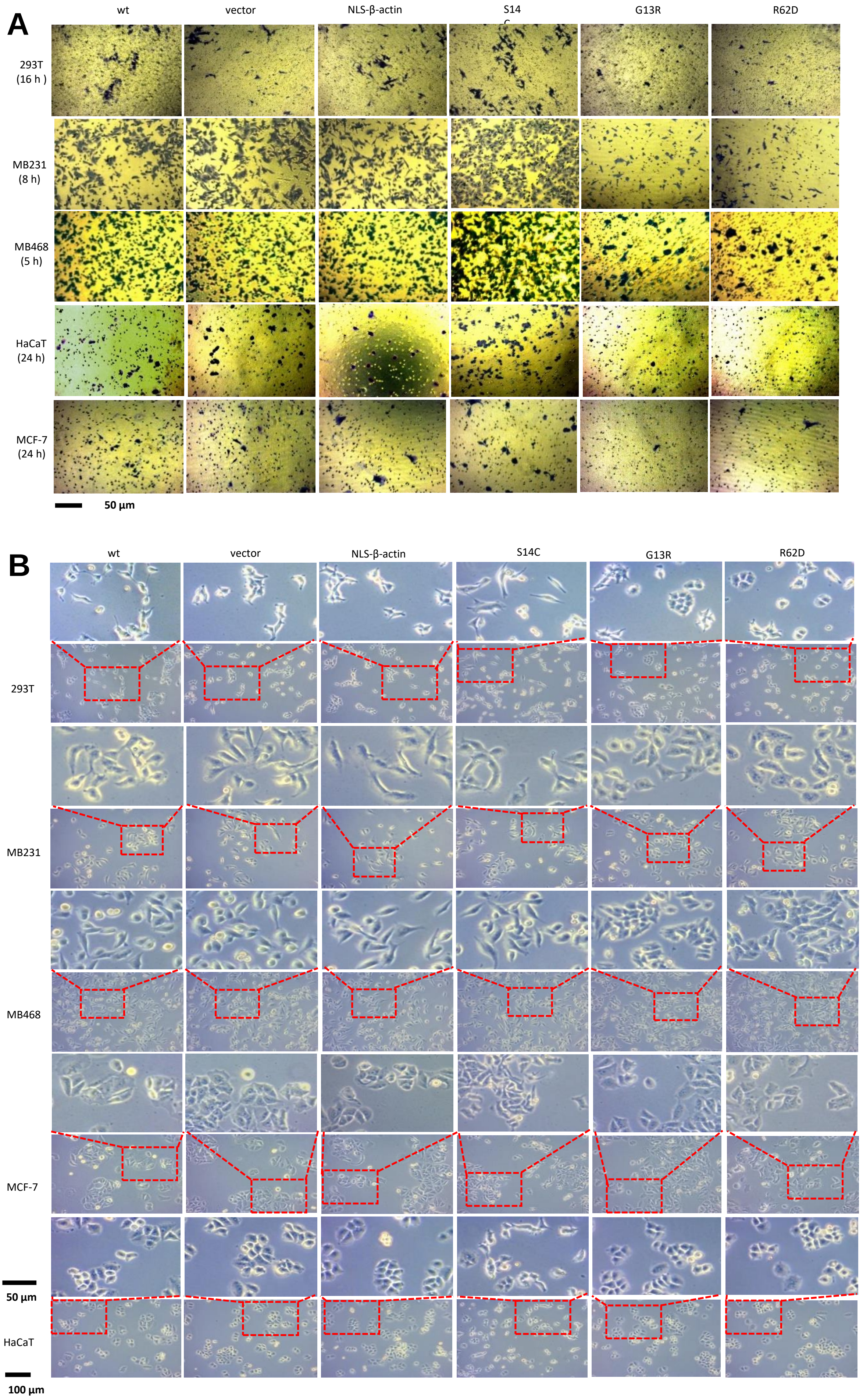

Fig S17. Overexpression of nuclear F/G-actin regulates cell migration and morphology.

A, HEK 293T, MDA-MB-231, MDA-MB-468, HaCaT and MCF-7 cells were transfected with the above-mentioned constructs and processed to chamber migration assays for indicated time points. Typical images showed that transfection with actin S14C enhanced cell migration, while transfection of G13R or R62D repressed cell migration.

B, The above transfected cells were cultured in basal medium for 24 h, showing that S14C transfected cells displayed elongated mesenchymal shape, while G13R or R62D transfected cells presented cuboidal epithelial structure.

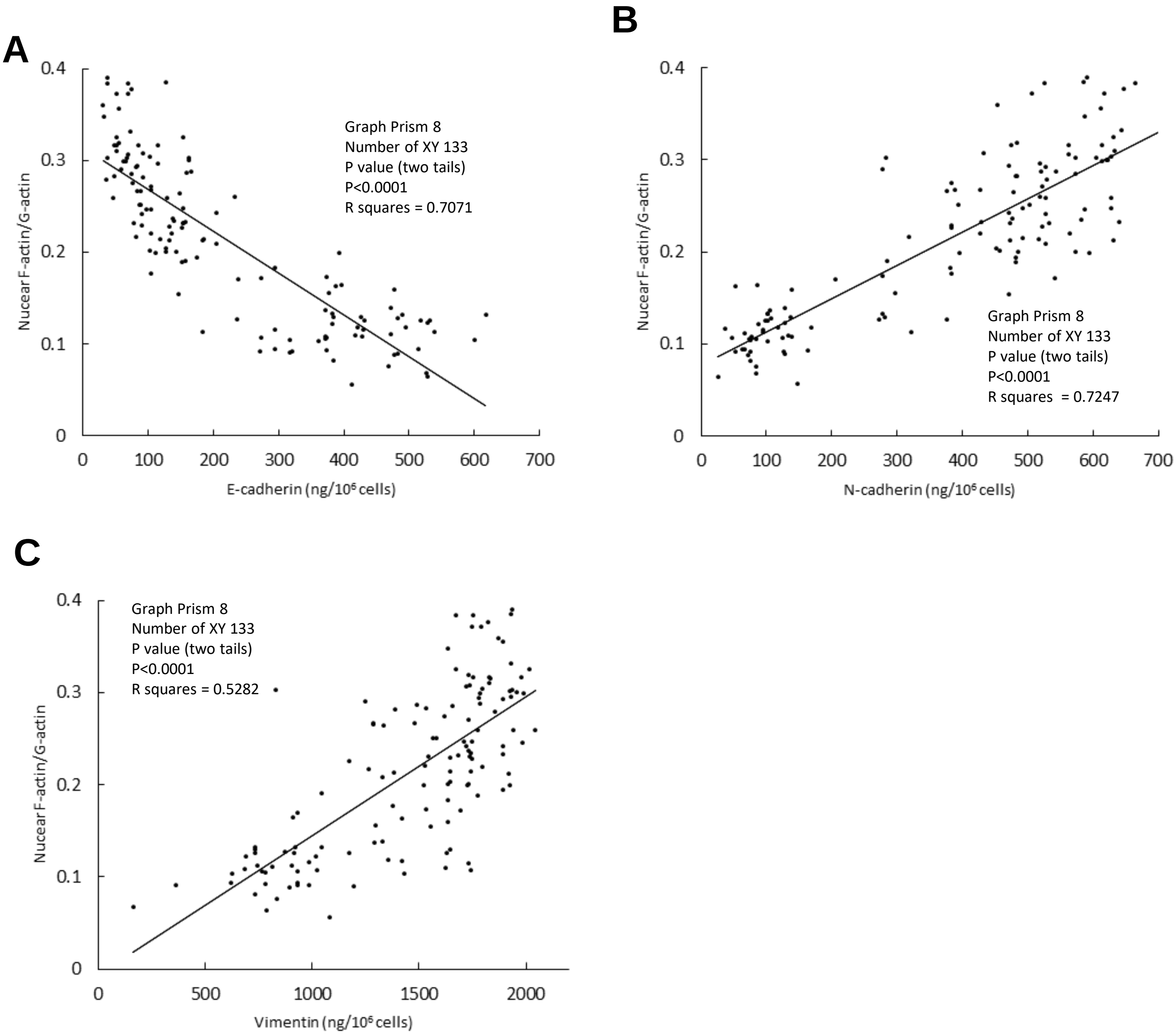

Fig S18. Nuclear F-actin/G-actin ratio is correlated with EMT in 133 cell lines.

A, The absolute values of E-cadherin, N-cadherin and vimentin and nuclear F-actin/G-actin protein levels in 133 cell lines were quantified by ELSA. The selected cell lines included: MDA-231-BoM-1833, 786-O, 4T1, 4T07, 66C14, 67NR, A431, A549, A2058, A2780, AC16, ACHN, ARH77, AU565, B16, BC3, BEAS-2B, BTH-1, BT20, BT474, BT549, BXPC3, C2C12, Caco-2, Caki-1, Caki-2, CI3K, CO-115, COLO-201, COLO-205, COLO-775, CW-9019, CRL-1476, Cos-1, Cos-7, CV-1, DLD-1, DU145, EMT6, ES2, FHC, H460, HaCaT, HCC1393, HCT6, HCT8, HCT15, HCT116, HDL100, HEK293T, Hela, Hep3B, HepG2, HEY, HGF, HL-1, HL-60, HLE, HT-1080, HT-29, HTB-123, HTB-126, Ho, Hs5787, Huh6, Huh7, ICE6, ICE18, JHH-1, Jurkat, JR75-1, JR-75-30, K652, KTC-1, LAPC-4, LAPC-9, Li7, LNCaP, MC3T3, MCF, MCF-7, MCF-10A, MDA-MB-157, MDA-MB-175, MDA-MB-231, MDA-MB-436, MDA-MB-468, MDA-MB-453, NIH3T3, NMuMG, PAN3, PANC-1, PC3, PC12, PLC/PRF/5, OV-2008, OVCAR-3, Raji, Rat2, RD, RFL-6, RH1, RH2, RH3, RH4, RH6, RH14, RH18, RH28, RH30, RIE-1, SK-NEP-1, SNU-16, SNU-378, SNU-449, Saos-2, SW48, SW480, SW620, SW837, SW1116, SW1353, T3M-4, T47V, T860, TOV-112D, SK-BR-3, UO-31, U87, U118, U343, U937 and YPEN-1. Pearson correlation analysis showed that F-actin/G-actin ratio was negatively correlated with E-cadherin protein levels in the cell lines. *$p<0.0001$, $n=133$, $R^2=0.7071$.*

B, Pearson correlation analysis showed that F-actin/G-actin ratio was positively correlated with N-cadherin protein levels in the cell lines. *$p<0.0001$, $n=133$, $R^2=0.7247$.*

C, Pearson correlation analysis showed that F-actin/G-actin ratio was positively correlated with vimentin protein levels in the cell lines. Trend line, R^2 and *p* values are labeled in the figures. *$p<0.0001$, $n=133$, $R^2=0.5282$.*

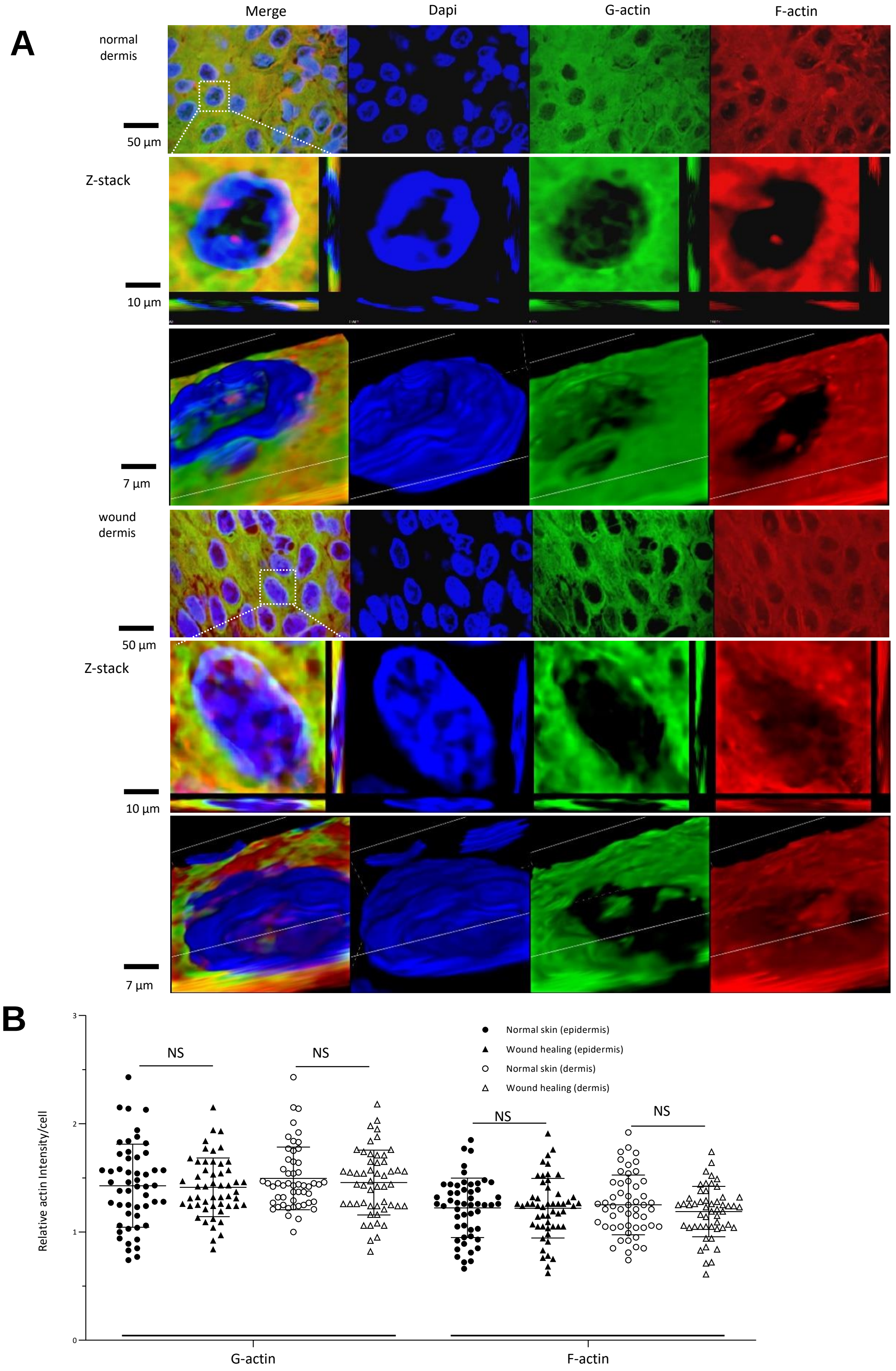

Fig S19. F/G-actin staining in mouse wound healing samples.

A, Typical z-stack images (xy, xz and yz projection and orthogonal view) showing the nuclear F-actin (Phalloidin staining, red) and G-actin (Deoxyribonuclease I staining, green) in wound healing and normal skin cells (dermis). Images for wound healing skins were randomly selected from central wound healing areas, while the normal skin images were randomly selected from the normal dermis areas far from the wound region.

B, ImageJ analysis showing the relative expression levels of F-actin and G-actin in the wound healing and normal skin cells. *$p<0.05$, **$p<0.01$ versus normal (*n=52*).
