# Supplementary material for: Nuclear Actin Polymerization Regulates Cell Epithelial‐Mesenchymal Transition
Source: Adv Sci (Weinh). 2023 Aug 11;10(28):2300425. doi: 10.1002/advs.202300425 (PMC10558697; doi:10.1002/advs.202300425)
Supplement: Supplementary file 2 — Supporting Information [file ADVS-10-2300425-s001.pdf]

ADVANCED SCIENCE
Open Access

Supporting Information

for *Adv. Sci.*, DOI 10.1002/advs.202300425

Nuclear Actin Polymerization Regulates Cell Epithelial-Mesenchymal Transition

*William W. Du, Javeria Qadir, Kevin Y. Du, Yu Chen, Nan Wu and Burton B. Yang**

Table S2. Sequences of primers and siRNAs

Primers

Primer Name	Sequence	Source / Repository
hu.E-cadherin-F	5' tcccatcagc tgcccagaaa atga	Eurofins Genomics LLC
hu. E-cadherin-R	5' gtgt ca gc tcctt ggcc ag tg atg	Eurofins Genomics LLC
hu.N-cadherin -F	5' gcgaatgatcttaggattggg	Eurofins Genomics LLC
hu.N-cadherin-R	5' gggaattcagcacccgcctc	Eurofins Genomics LLC
hu.Vimentin-F	5' cttctccgggagccagtccg	Eurofins Genomics LLC
hu.vimentin-R	5' cctgcggtaggaggacgagg	Eurofins Genomics LLC

siRNAs

siRNA Name	Sequence	Source / Repository
hu.mDia2-1	5' ccggcacaauucaguucaauu<br>3' uuggccguguuaagucaaguu	Gene Universal
hu.mDia2-2	5' cgggugccauaugaggaaauu<br>3' uugcccacgguauacuccuuu	Gene Universal
hu.EXP6-1	5' gcccucacagucguggaaauu<br>3' uucgggagugucagcaccuuu	Gene Universal
hu.EXP6-2	5' ccggguggugggagaaguuuuu<br>3' uuggcccaccaccucuucaaa	Gene Universal
hu.INP9-1	5' ccaucgccauuuuccuaaauu<br>3' uugguagcgguaaaaggauuu	Gene Universal
hu.INP9-2	5' cccaggaccuacuggcaaauu<br>3' uuggguccuggaugaccguuu	Gene Universal
hu.MYBBP1A-1	5' gcccuggcccaguaccaaauu<br>3' ggcgggaccgggucaugguuu	Gene Universal
hu.NKRF-1	5' gcuguccaaaccuuccaaauu<br>3' ggcgacagguuuggaagguuu	Gene Universal
hu.MYPOP-1	5' gcgggcgaagcggaggaaauu<br>3' cccgcccgcuucgccuccuuu	Gene Universal
hu.MYPOP-2	5' ccccacacaagcggagaaauu<br>3' ccgggguguguucgccucuuu	Gene Universal
hu.α-catenin-1	5' ggucacaggcauuuccaauuu<br>3' ggccaguguccguaaagguua	Gene Universal
hu.FLNA-1	5' ccgccaauaacgacaagaauu<br>3' ccggcgguuauugcuguucuu	Gene Universal
Hu.PFN-1	5' ggguguccugguuggcaaauu<br>3' ggcccacaggaccaaccguuu	Gene Universal

DOI [to be added]
